# The polymeric glyco-linker controls the signal outputs for plasmonic gold nanorod biosensors due to biocorona formation†

**DOI:** 10.1039/d1nr01548f

**Published:** 2021-06-03

**Authors:** Alessia Pancaro, Michal Szymonik, Panagiotis G. Georgiou, Alexander N. Baker, Marc Walker, Peter Adriaensens, Jelle Hendrix, Matthew I. Gibson, Inge Nelissen

**Affiliations:** Health Unit, Flemish Institute for Technological Research (VITO) Boeretang 200 Mol BE-2400 Belgium inge.nelissen@vito.be; Dynamic Bioimaging Lab, Advanced Optical Microscopy Centre and Biomedical Research Institute, Hasselt University Agoralaan C Diepenbeek BE-3590 Belgium; Department of Chemistry, University of Warwick Gibbet Hill Road Coventry CV4 7AL UK m.i.gibson@warwick.ac.uk; Department of Physics, University of Warwick Gibbet Hill Road Coventry CV4 7AL UK; Applied and Analytical Chemistry, Institute for Materials Research, Hasselt University Agoralaan D Diepenbeek BE-3590 Belgium; Warwick Medical School, University of Warwick Gibbet Hill Road Coventry CV4 7AL UK

## Abstract

Gold nanorods (GNRs) are a promising platform for nanoplasmonic biosensing. The localised surface plasmon resonance (LSPR) peak of GNRs is located in the near-infrared optical window and is sensitive to local binding events, enabling label-free detection of biomarkers in complex biological fluids. A key challenge in the development of such sensors is achieving target affinity and selectivity, while both minimizing non-specific binding and maintaining colloidal stability. Herein, we reveal how GNRs decorated with galactosamine-terminated polymer ligands display significantly different binding responses in buffer compared to serum, due to biocorona formation, and how biocorona displacement due to lectin binding plays a key role in their optical responses. GNRs were coated with either poly(*N*-(2-hydroxypropyl)methacrylamide) (PHPMA) or poly(*N*-hydroxyethyl acrylamide) (PHEA) prepared *via* reversible addition–fragmentation chain-transfer (RAFT) polymerisation and end-functionalised with galactosamine (Gal) as the lectin-targeting unit. In buffer Gal-PHEA-coated GNRs aggregated upon soybean agglutinin (SBA) addition, whereas Gal-PHPMA-coated GNRs exhibited a red-shift of the LSPR spectrum without aggregation. In contrast, when incubated in serum Gal-PHPMA-coated nanorods showed no binding response, while Gal-PHEA GNRs exhibited a dose-dependent blue-shift of the LSPR peak, which is the opposite direction (red-shift) to what was observed in buffer. This differential behaviour was attributed to biocorona formation onto both polymer-coated GNRs, shown by differential centrifugal sedimentation and nanoparticle tracking analysis. Upon addition of SBA to the Gal-PHEA coated nanorods, signal was generated due to displacement of weakly-bound biocorona components by lectin binding. However, in the case of Gal-PHPMA which had a thicker corona, attributed to lower polymer grafting densities, addition of SBA did not lead to biocorona displacement and there was no signal output. These results show that plasmonic optical responses in complex biological media can be significantly affected by biocorona formation, and that biocorona formation itself does not prevent sensing so long as its exact nature (*e.g.* ‘hard *versus* soft’) is tuned.

## Abstract 2

Glycosylated gold nanorods are shown to have distinct binding responses in buffer, compared to serum, due to the formation of a biocorona which is displaced during lectin binding.

## Graphical abstract

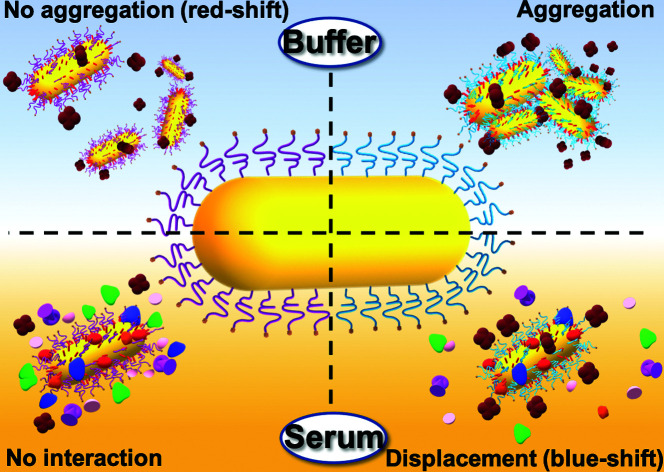

## Introduction

Spherical gold nanoparticles (GNPs) are one of the most widely studied nanomaterials. Their established and versatile synthetic methods, biocompatibility, ease of surface functionalisation and unique optical properties^1,2^ have facilitated a wide range of applications in diagnostics, therapeutics and drug delivery.^3–6^ The optical properties of GNPs are dominated by the phenomenon of localised surface plasmon resonance (LSPR), a collective oscillation of electrons in resonance with incident light, strongly localised to the GNPs surface.^7,8^ The wavelength of this oscillation is dependent on the size of the particle and the refractive index of the surrounding medium. This property has been exploited to produce biosensor devices, as the binding of biomolecules to the GNP's surface leads to changes in the LSPR peak, which are easily measured.^9,10^ Many colorimetric assays, for chemical and biological sensing applications, have been developed based on, for example, aggregation, etching, growth and nanozymes.^11^ For example, a colorimetric assay using glyco-GNPs have been reported for the detection of lectins, cholera toxin and influenza viruses.^12–14^

Changes in the nanoparticle shape affect a GNR's plasmonic properties, which can be advantageous for biosensing applications. The anisotropy of gold nanorods (GNRs) leads to a splitting of the optical absorption bands into two separate LSPR peaks corresponding to resonances along the short and long axes of the rod, termed the transverse and longitudinal bands respectively. The longitudinal band is typically more sensitive to local refractive index changes than the transverse peak.^15^ Importantly, and in contrast to spherical GNPs, the absorbance maximum of this peak is dependent on the rod aspect ratio and can be fine-tuned from visible to near-infrared (*λ* ∼ 600 to >1300 nm), where biological tissues exhibit the highest optical transparency.^10,16–18^ This allows measurement directly in complex biological fluids such as blood, enabling a broad range of *in vitro* and *in vivo* applications.^19^ Therefore, GNR-based LSPR sensors represent an important advancement for rapid, simple, label-free, and sensitive detection of low target molecule concentrations.^20–23^ For example, lactose-functionalised GNRs have been fabricated as efficient biosensors to detect the cancer biomarker Galectin-1 at concentrations below 10^−13^ M.^24^ Lipid-capped GNRs have been used for the label-free detection of a lipophilic drug in aqueous solution and a lipopeptide in serum.^25^ GNRs can also provide signal enhancement in chip-based SPR detection. For example, Law *et al.* have shown a 40-fold sensitivity enhancement for the detection of tumour necrosis factor alpha (TNF-α) when using antibody-conjugated GNRs as a plasmonic coupling partner.^26^ Compared to other plasmonic nanostructures, gold nanorods show high plasmonic surface sensitivity due to their high aspect ratio.^27^

To enable LSPR-based biosensing, the nanoparticles must be decorated with recognition units tethered to the surface by a ligand. In this work, we focused on the detection of lectins, which are glycan-binding proteins that play a key role in many biological processes, including cell–cell adhesion, cell recognition, cell differentiation, and infection by pathogens.^28–30^ Lectins interact only weakly (millimolar *K*_d_ values) with individual monosaccharides, however multivalent interactions result in *K*_d_ values in the nanomolar range towards their glycan targets.^31–35^ The multivalent presentation of the same glycan compensates for the low lectin–monosaccharide affinity, leading to a non-linear increase in binding affinity, termed the “cluster glycoside effect”.^32,36^

A broad range of surface ligands including peptides,^37^ polymers,^38^ and cationic thiolated ligands^39^ among others,^40^ have been used to stabilize nanoparticles and anchor functional groups. Using polymeric ligands, Gibson and co-workers have demonstrated the importance of tuning the polymer length to achieve the crucial balance between stability and aggregation of glyco-nanoparticles upon lectin binding.^41–44^ In addition, ligands play a key role in controlling particle surface properties, such as charge and chemical reactivity, which drive a particle's interactions with bio(macro)molecules. Exposure of nanoparticles to complex biological media leads to the formation of a biomolecular corona, termed a “biocorona”.^45–49^ The biocoronas initially consist of the most abundant and fast-diffusing molecules in solution, before being partially replaced over time by proteins that have a higher affinity for the nanoparticle surface. These higher affinity proteins can act to mask the ligands displayed on the nanoparticles, an hence interfere with their recognition capability.^50,51^ For example, transferrin coated nanoparticles have been shown to loose their targetting capacity due to protein corona formation.^52^ Understanding the dynamic exchange rates between bound and unbound proteins on nanoparticles in biological systems is vital for the design of new nanoparticle-based biosensors capable of detecting target molecules directly in the physiological environment.^53–56^

Herein, we study the sensing performance of GNRs coated with galactosamine-modified polymeric linkers for the detection of the lectin soybean agglutinin (SBA), used as a model analyte. We observe how the structure of surface tethered glycopolymers controlled the magnitude and reversibility of serum–biomolecule binding to the nanoparticles and explore the impact of this on biosensing in serum, compared to simple buffers. It is found that careful selection of the polymeric linker promotes the formation of a reversible biocorona, which can be displaced upon lectin binding, generating signal. This shows that biocorona formation in complex media does not fundamentally prevent gold nanorod biosensing and that macromolecular engineering of the polymer tethers can be used to optimise device performance.

## Experimental

### Materials

All reagents were used as supplied, unless otherwise stated. Citrate-stabilised gold nanorods (GNRs) of 10 nm width and 38 nm length (*λ*_max_ = 780 nm) were purchased from Nanopartz. Human serum (H6914), d-(+)-galactosamine hydrochloride (99%), HEPES buffer, NaCl, CaCl_2_, triethylamine (>99%), 2-(dodecylthiocarbonothioylthio)-2-methylpropionic acid pentafluorophenyl ester (98%, PFP-DMP), and monomers *N*-(2-hydroxypropyl)methacrylamide (99%, HPMA) and *N*-hydroxyethyl acrylamide (97%, HEA) were all purchased from Sigma-Aldrich. MnCl_2_ was purchased from VWR. Soybean agglutinin (SBA) lectin was obtained from Vector Laboratories. Photo-polymerisation reactions were conducted using a blue LED strip light (3 meters with 180 LEDs, *λ* = 460–465 nm). All experiments were conducted using Milli-Q grade water (resistivity of 18.2 mΩ cm at 25 °C, 4 ppb total organic carbon).

### Methods

#### Polymer synthesis and glycan modification

The synthesis and characterisation of PFP-PHPMA (pentafluorophenyl ester-poly(*N*-(2-hydroxypropyl) methacrylamide)) and PFP-PHEA (pentafluorophenyl ester-poly(*N*-hydroxyethyl acrylamide)) with a range of degrees of polymerisations (40, 50, 55, 68 for PFP-PHPMA and 26, 35, 50, 60 for PFP-PHEA), as well as end-group modification using galactosamine (Gal) were performed according to previously reported experimental protocols (see ESI† for detailed synthetic procedure).^57^

#### (Glyco)polymer conjugation onto gold nanorods surface

Approximately 2 mg of each (glyco)polymer was dissolved in 200 μL of water and mixed by pipetting with 800 μL of GNR suspension at 5 OD. After a 30 min incubation at room temperature in the dark, the particles were sonicated for 1 min using an ultrasonic bath at 40 kHz (Branson CPX1800H), centrifuged at 12 000*g* and 20 °C for 10 min, and the supernatant removed. This was followed by 3 cycles of re-suspension in 1 mL water, centrifugation and decanting. A Sigma 3-30KS centrifuge was used with 1.5 mL volume tubes for all preparative centrifugation. The particles were finally resuspended in 1 mL water (OD = 4) and stored in polypropylene graduated tubes at 4 °C until use.

#### Saline stability-induced aggregation studies

A solution of 1 M NaCl was serially diluted down to 0.031 M in clear, flat-bottomed 384-well NUNC plates and used for all optical measurements. 3 μL of citrate-GNRs and conjugated GNRs were added to each well, mixed and incubated at room temperature for 30 min, and then an absorbance spectrum was recorded from 400 nm to 1000 nm with 1 nm resolution.

#### Lectin binding studies by absorbance

A stock solution of 1 mg mL^−1^ SBA was prepared in 10 mM HEPES buffer (pH 7.5) containing 50 mM NaCl, 0.1 mM CaCl_2_ and 0.01 mM MnCl_2_. Functionalised GNRs (3 μL, yielding ∼0.3 final OD) were added to a series of different SBA concentrations (10–100 μg mL^−1^) in HEPES buffer for a final volume of 40 μL. The plate was gently agitated at room temperature for 30 min, and then absorbance spectra were recorded every 30 min over 2 hours from 400 to 999 nm with 1 nm interval using a Biotek Synergy HT microplate reader.

For the assay carried out in serum, lyophilised SBA was dissolved in human serum at 1 mg mL^−1^ and diluted in serum (10–100) μg mL^−1^. A CLARIOstar Plus plate reader (BMG Labtech) was used to record spectra every 5 min, with 30 seconds of plate shaking prior to each measurement.

#### (Glyco)polymer-coated GNRs studies in serum

(Gal)-PHEA_35_ coated GNRs (300 μL) were added to 1.5 mL of human serum in a 2 mL Eppendorf Protein LoBind tube and incubated at room temperature for 2 hours on a shaker at 40 rpm to allow formation of a biocorona on the particles’ surface. The GNRs suspension was centrifuged at 12 000*g* for 10 min at 20 °C, the supernatant was removed and the pellet washed three times by pipetting in 1 mL of 10 mM HEPES buffer. The particles were finally resuspended in 300 μL HEPES buffer and 40 μL volumes transferred to the wells of a 384-well plate. UV-Vis spectra were recorded every 5 min. After 1 hour, 4 μL of 1 mg mL^−1^ SBA was injected to a final concentration of 100 μg mL^−1^ and the response monitored for a further 3 hours. To quantify the thickness of the biocorona, Gal-PHPMA_40_ and (Gal)-PHEA_35_ coated GNRs were analysed directly in serum (without washing) using differential centrifugal sedimentation (DCS) as described below.

### Characterisation techniques

#### NMR spectroscopy

Proton (^1^H-NMR) and fluorine (^19^F-NMR) nuclear magnetic resonance spectra were recorded at 300 MHz or 400 MHz on a Bruker DPX-300 or DPX-400 spectrometer respectively, with methanol-*d*_4_ as the solvent. Chemical shifts of protons are reported as *δ* in parts per million (ppm). Alternatively, NMR spectra were recorded at room temperature on a Varian/Agilent Inova 400 MHz spectrometer using a 5 mm four-nucleus pulsed field gradient (PFG) probe. For ^1^H-NMR, the chemical shift scale (*δ*) in ppm was calibrated relative to methanol-*d*_4_ (3.31 ppm), while CFCl_3_ (0 ppm) was used for ^19^F-NMR. For ^1^H-NMR (^19^F-NMR), free induction decays were collected with a 90° pulse of 6.8 (8.0) μs, a spectral width of 6.6 (21) kHz, an acquisition time of 3.5 (0.5) s, a preparation delay of 12 (1) s and 32 (400) accumulations (scans). A line-broadening factor of 0.3 Hz (^1^H-spectra) or 5 Hz (^19^F-spectra) was applied before Fourier transformation to the frequency domain.

#### FT-IR spectroscopy

Fourier Transformed-Infrared (FT-IR) spectroscopy measurements were carried out in the range of 650 to 4000 cm^−1^ using a Cary 630 FT-IR spectrometer (Agilent) or Nicolet iS10 spectrometer (Thermo Fisher Scientific) in the range of 4000 to 400 cm^−1^.

#### Size exclusion chromatography (SEC)

SEC analysis was performed on an Infinity II MDS instrument (Agilent) equipped with differential refractive index (DRI), viscometry (VS), dual angle light scatter (LS) and variable wavelength UV detectors. The system was equipped with 2 × PLgel Mixed D columns (300 × 7.5 mm) and a PLgel 5 μm guard column. The mobile phase used was dimethylformamide (DMF) HPLC grade containing 5 mM NH_4_BF_4_ at 50 °C at flow rate of 1.0 mL min^−1^. Poly(methyl methacrylate) (PMMA) standards (Agilent EasyVials) were used for calibration between 555–955 000 g mol^−1^. Analyte samples were filtered through a nylon membrane with 0.22 μm pore size before injection. Number average molecular weights (*M*_n_), weight average molecular weights (*M*_w_) and dispersities (*Đ*_M_ = *M*_w_/*M*_n_) were determined by conventional calibration using Agilent SEC software.

#### UV-Visible spectroscopy

UV-Vis absorption spectra were acquired at room temperature (25 °C) on a CLARIOstar Plus spectrophotometer. All absorbance spectra were recorded between *λ* = 400–1000 nm with 1 nm resolution and 30 seconds of plate shaking at 100 RPM applied before each measurement. Results were smoothed using a Savitzky–Golay filter (order 4, window width 31). Peak maxima were determined from the zero crossings of the derivative of the smoothed data. All measurements were performed with at least two replicates (*n* ≥ 2).

**Differential centrifugal sedimentation** (DCS) was performed to assess binding of glycopolymers, serum molecules and SBA on the GNR surface by measuring the particles’ size distribution. For this a CPS DC24000 disc centrifuge was used with a 8–24% (w/w) sucrose gradient and a rotation speed of 24 000 RPM. For measurements in serum, a fresh sucrose gradient was prepared for each measurement. Before each run, polyvinyl chloride latex beads (239 nm) with narrow size distribution are used as calibration standard to ensure accuracy of the measurements. All the measurements were performed with at least two replicates (*n* ≥ 2). As the settling of particles is shape-dependent, for GNRs a ‘non-sphericity factor’ (NSF) of 2.85 was applied in the CPS software. The binding of biomolecules onto the GNRs’ surface increases the particles’ size, but lowers their overall density. The CPS analysis assumes a constant particle density, so over-estimating the particle density means an under-estimate of the particle size.^58,59^ For this reason, the binding of polymers or biomolecules to the GNRs results in an apparent decrease in the particle size reported by CPS. A core–shell mathematical model was used to analyse the coating thickness of the GNRs as previously described.^60,61^

**Nanoparticle tracking analysis** (NTA) was performed to measure GNR sizes before and after functionalisation using a NanoSight NS500 instrument in scatter mode with a laser output of 75 mW at 532 nm (green) and sCMOS camera (camera level set at 15). All the samples were analysed in duplicate at 25 °C and 3 videos of 60 seconds were recorded (1499 frames with 25 frames per second) for each sample. The number of particles/frame ranged from 30 to 90 for the GNR samples, and none were detected in the buffer control. The samples were diluted to 10^8^–10^9^ particles per mL in MilliQ water, or 0.22 μm (Millex-GV) filtered HEPES buffer for serum-incubated samples that were washed in buffer. For calibration, 100 nm polystyrene (PS) microspheres were used. The mode was derived from a particle number concentration-based size distribution using the NTA software version 3.0.

**ζ-Potential** was measured on a ZetaView-Twin instrument (Particle Metrix). Alumina zeta potential standard was used and all samples were measured in 11 positions at 22 °C in triplicate. Zeta potential was calculated from the corresponding electrophoretic mobilities (*μ*_E_) by using Henry's correction of the Smoluchowski equation (*μ*_E_ = 4π*ε*_0_*ε*_r_*ζ*(1 + *κr*)/6π*μ*), where *ε*_0_ is the dielectric permittivity of the vacuum, *ε*_r_ is the relative permittivity of the liquid, ζ is the zeta potential, *κ* is the Debye length, *r* is the particle radius and *μ* is the mobility.

**Dynamic light scattering (DLS)** was measured on a Zetasizer ZS (Malvern Panalytical). Measurements were carried out using a 4 mW He–Ne 633 nm laser module operating at 25 °C at an angle of 173° (back scattering), and results were analysed using Malvern DTS 7.03 software. All determinations were repeated in duplicate with at least 5 measurements recorded for each run.

#### Transmission electron microscopy

Dry-state stained TEM imaging was performed on a JEOL JEM-2100Plus microscope operating at an acceleration voltage of 200 kV. All dry-state samples were diluted with deionised water and then deposited onto formvar-coated copper grids.

#### X-ray photoelectron spectroscopy (XPS)

The samples were attached to electrically-conductive carbon tape, mounted onto a sample bar and loaded into a Kratos Axis Ultra DLD spectrometer which possesses a base pressure below 1 × 10^−10^ mbar. XPS measurements were performed in the main analysis chamber, with the sample being illuminated using a monochromated Al Kα X-ray source. The measurements were conducted at room temperature and at a take-off angle of 90° with respect to the surface parallel. The core level spectra were recorded using a pass energy of 20 eV (resolution approx. 0.4 eV), from an analysis area of 300 μm × 700 μm. The spectrometer work function and binding energy scale were first calibrated using the Fermi edge and 3d_5/2_ peak recorded from a polycrystalline Ag sample. In order to prevent surface charging, the surface was flooded with a beam of low energy electrons throughout the experiment and this necessitated recalibration of the binding energy scale. To achieve this, the C–C/C–H component of the C 1*s* spectrum was referenced to 285.0 eV. The data were analysed in the CasaXPS package, using Shirley backgrounds and mixed Gaussian–Lorentzian (Voigt) lineshapes. For compositional analysis, the analyser transmission function has been determined using clean metallic foils to determine the detection efficiency across the full binding energy range.

## Results and discussion

### Polymer synthesis

Two water soluble, non-ionic polymers, poly(*N*-(2-hydroxypropyl) methacrylamide) (PHPMA) and poly(*N*-hydroxyethyl acrylamide) (PHEA), were synthesised by photo-initiated RAFT polymerisation to maximise end-group fidelity. These polymers have previously been used to functionalise spherical gold nanoparticles, where subtle structural differences were found to produce large changes in grafting density and responses to analyte binding.^57^ Each polymer was prepared with four different degrees of polymerisation (DP = 40, 50, 55, 68 for PHPMA and DP = 26, 35, 50, 60 for PHEA) by tuning the feed ratio. Polymers were characterised using ^1^H-NMR and SEC (Fig. S1 and S2, ESI†). Narrow monomodal molecular weight distributions were observed with low dispersity values (*Đ*_M_ ≤ 1.3) in all cases indicating a controlled photo-polymerisation (Table 1). DP was also assessed by ^1^H NMR end-group analysis in methanol-*d*_4_ showing *M*_n_ values lower than that found from SEC, as has been previously reported.^57^ Polymer DP (in subscript) referred to from here is from ^1^H NMR. Retention of the pentafluoro phenyl (PFP) end-group during polymerisation was confirmed *via*^19^F NMR (Fig. S3, ESI†) and FT-IR analysis. Galactosamine was installed at the end-groups for both PHEA/PHPMA homopolymers displacement of the PFP end-group at the α-terminus,^62^ and confirmed by ^19^F-NMR and FT-IR analysis before and after modification (Fig. S3 and S4, ESI†).

**Table 1:** Polymers synthesised by photo-RAFT

Polymer^a^ (—)	[M] : [CTA]^b^ (—)	Conversion^c^ (%)	*M*_n, NMR_ d (g mol^−1^)	SEC analysis	
				*M*_n, SEC_ e (g mol^−1^)	*Đ*_M_ e
PHPMA_40_	40	100	6300	9600	1.17
PHPMA_50_	60	83	7700	11 300	1.22
PHPMA_55_	80	69	8400	12 500	1.19
PHPMA_68_	100	68	10 300	15 500	1.25
PHEA_26_	120	22	3500	8200	1.13
PHEA_35_	140	25	4600	9800	1.12
PHEA_50_	160	31	6300	14 500	1.13
PHEA_60_	180	33	7400	16 500	1.11

a Polymer names are determined according to the average degree of polymerisation (DP) determined by ^1^H-NMR end-group analysis in methanol-*d*_4_.

b [M] : [CTA] indicates the ratio between monomers (M) and the chain transfer agents (CTA).

c Monomer conversion (%) calculated by comparing the integration values of the monomer signals with those of the corresponding signals of the polymer.

d *M*_n, NMR_ was calculated by end-group analysis by comparing the integrations of the –CH_3_ signals (*δ* 0.92 ppm) of dodecyl end-group with those of the corresponding signals of the polymer backbone.

e *M*_n_ and *Đ*_M_ values calculated from PMMA standards using 5 mM NH_4_BF_4_ in DMF as the eluent.

### Conjugation of glycopolymers onto gold nanorods

**Scheme 1:**
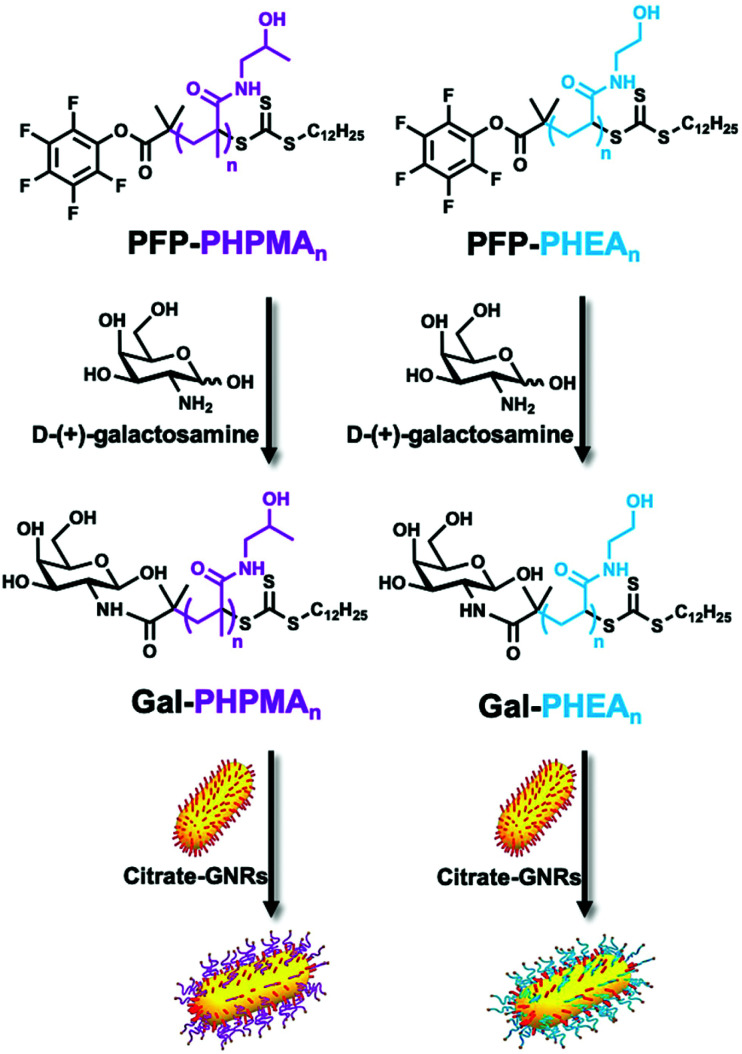
Schematic of end-group modification of PFP-PHPMA (left) and PFP-PHEA (right) telechelic homopolymers of different chain lengths (*n*) using galactosamine, followed by functionalisation of citrate-stabilised gold nanorods. Note, RAFT agent cleavage can occur during functionalisation depending on excess used, but does not affect GNR immobilisation.

Citrate-stabilised GNRs were mixed with thiocarbonylthio-terminated polymers (PHPMA_*n*_ and PHEA_*n*_) or thiocarbonylthio-terminated glycopolymers (Gal-PHPMA_*n*_ and Gal-PHEA_*n*_, Scheme 1) of various lengths (*n*) to produce a library of (glyco)polymer-coated GNRs (termed (Gal)-PHPMA GNRs and (Gal)-PHEA GNRs). Excess of polymer was removed by multiple centrifugation and resuspension cycles.

**Fig. 1:**
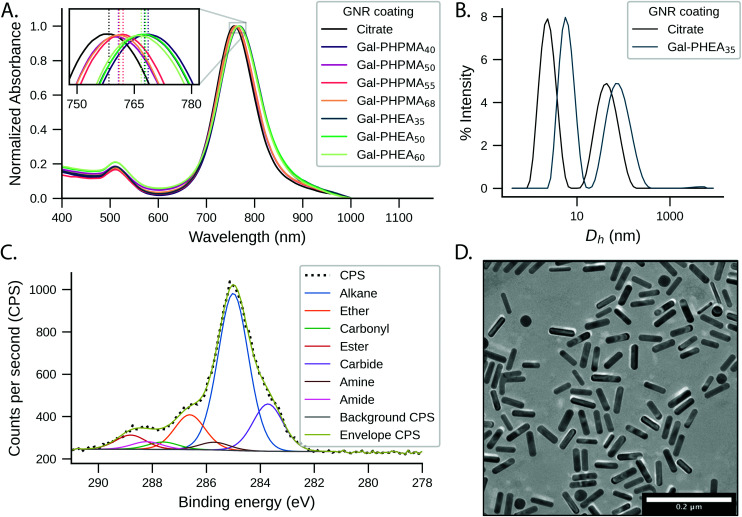
Glyconanoparticle characterisation. (A) Representative example of UV-Vis absorption spectra of GNRs coated with Gal-PHPMA and Gal-PHEA of different lengths. Inset: zoomed view on the LSPR peak bands; (B) intensity-weighted DLS size distributions of Gal-PHEA_35_ GNRs compared to pristine GNRs; (C) XPS C 1*s* characterisation of Gal-PHEA_35_ GNRs and (D) representative dry-state TEM image of Gal-PHEA_35_ GNRs.

The physico-chemical properties for the glycopolymer-coated GNRs and unmodified GNRs were analysed by UV-Vis, ζ-potential, XPS, TEM, DLS, DCS and NTA (Fig. 1, Tables S1 and S2 and Fig. S5–S7, ESI†). All functionalised nanorods were colloidally stable in aqueous solution apart from those conjugated with the shortest PHEA_26_ (or Gal-PHEA_26_) which led to macroscopic precipitation (data not shown) and were not taken further in this study. UV-Vis spectroscopy revealed a red-shift of the longitudinal LSPR band (Fig. 1A) for the glycopolymer coated GNRs, while DLS (Fig. 1B) and ζ-potential (Table S1, ESI†) confirmed the successful attachment of the glycopolymers to the particle surface. An example TEM is shown in Fig. 1D showing the nanorod dimensions were retained (*i.e.* no ripening of particles) during coating. Differential centrifugal sedimentation (DCS) and nanoparticle tracking analysis (NTA) also supported an increase in particle size following polymer addition (see Table S1, ESI†).

To further confirm the presence of glycopolymers on the surface of the GNRs, X-ray photoelectron spectroscopy (XPS) analysis was performed on Gal-PHPMA_40_ and Gal-PHEA_35_ coated GNRs (Fig. 1C and Fig. S5 and S6, ESI†). The presence of N 1*s* peaks, which are not present on the naked particles (Fig. S7, ESI†) or found commonly in background contaminants, confirmed successful binding of the polymers onto the particle surface (Table S2, ESI†). The ratio of N 1*s* : Au 4*f* peak areas was higher for PHEA_35_-GNRs *versus* PHPMA_40_, indicating a higher surface grafting density for the PHEA polymer.^57^ Note, the impact of grafting density is discussed later in this article.

One prerequisite for any biomedical application of nanoparticles is their colloidal stability under physiological conditions, and in particular due to saline which is present at ∼0.150 M (NaCl). We tested the stability of the conjugated gold nanorod suspensions by titrating NaCl and using UV-Vis to monitor aggregation. Citrate-GNRs aggregated above 0.125 M NaCl, while all Gal-PHPMA GNRs (DP 40, 50, 55, 68) and Gal-PHEA GNRs (DP 35, 50, 60) remained as stable dispersions up to 1 M NaCl (Fig. S8, ESI†). The increased colloidal stability is essential for sensing applications to avoid false-positive read-outs and also provides additional evidence for the polymer coating providing steric stabilisation.

### Lectin binding studies in buffer

The plant lectin soybean agglutin (SBA) was employed as a model system to study the lectin binding behaviour of various glycopolymer-coated GNRs. SBA has high affinity for *N*-acetylgalactosamine (GalNAc).^63–65^ When conjugated to the polymers *via* an amide bond, galactosamine acts as a structural mimic of GalNAc.^57^ The particles were incubated with SBA concentrations between 0 and 100 μg mL^−1^ in 10 mM HEPES buffer and UV-Vis spectra recorded to observe changes in the LSPR peaks. Wheat germ agglutinin (WGA) which exhibits low affinity towards GalNAc,^66^ and particles with non-glycosylated polymer were used as negative controls.

**Fig. 2:**
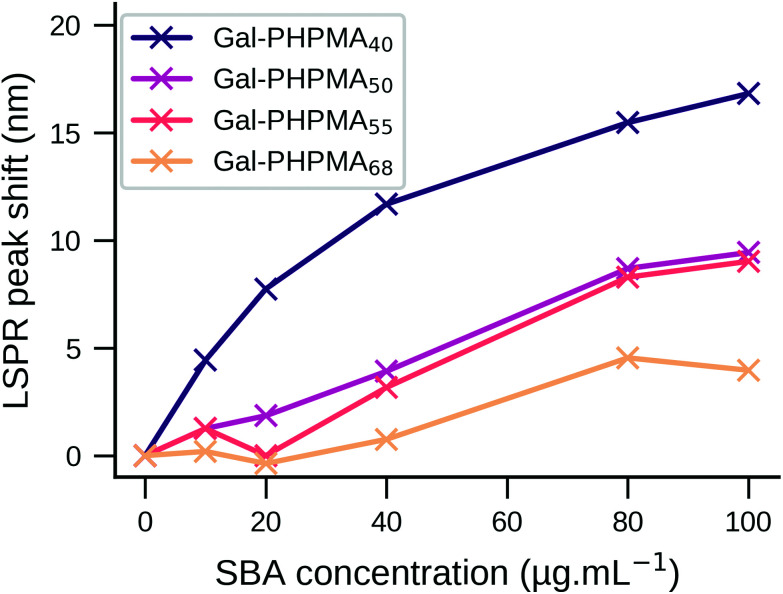
LSPR peak shift of Gal-PHPMA GNRs as a function of SBA concentration in buffer. *Y*-Axis shows the LSPR wavelength red-shift as determined by UV-Vis spectroscopy after 2 hours of incubation. Different PHPMA polymer lengths (DP = 40, 50, 55, 68) were compared.

Despite only minor structural differences, the behaviour for the two polymers upon analyte addition was markedly different. Addition of SBA to Gal-PHEA GNRs (DP 35, 50, 60) led to cross-linking and aggregation with shorter linker lengths as seen by broadening of the LSPR peak and a decrease in the overall absorbance (Fig. S9/S10, ESI†)^67^ – this was also visible to the naked eye. No change in the spectrum was observed with WGA (wheat germ agglutinin, which has no affinity to GalNAc) or using non-glycosylated polymers (Fig. S11/S12, ESI†). The same assay carried out with Gal-PHPMA GNRs (DP 40, 50, 55, 68) produced no aggregation, but instead an SBA concentration-dependent red-shift of the LSPR peak which is attributed to the local refractive index changes at the surface due to the binding interaction. Moreover, increasing the linker length led to a decrease in the assay sensitivity, as can be seen by the smaller LSPR peak shift (Fig. 2), which would be consistent with increasing distance of the binding event from the rod surface.^68,69^ Again, negative controls of WGA and non-glycosylated particles showed no changes, as would be expected (Fig. S12, ESI†).

GNRs incubated with 100 μg mL^−1^ SBA were further studied by DLS. For Gal-PHEA_35_ GNR there was clear aggregation in agreement with the UV-Vis data (Fig. S13†) but there was no aggregation for Gal-PHPMA_40_ GNR, although a small size increase was seen. The different responses can be attributed to the PHPMA ligands having lower grafting densities (and hence fewer glycans) on the rod surface (as shown by XPS, above) compared to PHEA, and provides further evidence that an absence of aggregation does not always indicate an absence of binding between different multivalent systems.^57^ Together, this data shows that by carefully tuning the polymer tether, the outcome of plasmonic bioassays towards either aggregation or LSPR shifts can be fine-tuned, which is a valuable tool in the design of nanoparticle biosensors.

### Lectin binding studies in serum

The ability to use glycosylated PHPMA coated GNRs to target lectins, but without any unwanted aggregation could be appealing for *in vivo* or *ex vivo* biosensing, where aggregation is undesirable. However, the behaviour predicted from buffer (above) ignores the contribution and impact of other competing bio(macro)molecules which are present in many ‘real’ samples such as liquid biopsies. For this reason we evaluated SBA binding towards Gal-PHPMA GNRs and Gal-PHEA GNRs in serum. The stability of nanoparticles in such a complex environment is generally considered to be provided by the formation of the biomolecular corona.^60^

**Fig. 3:**
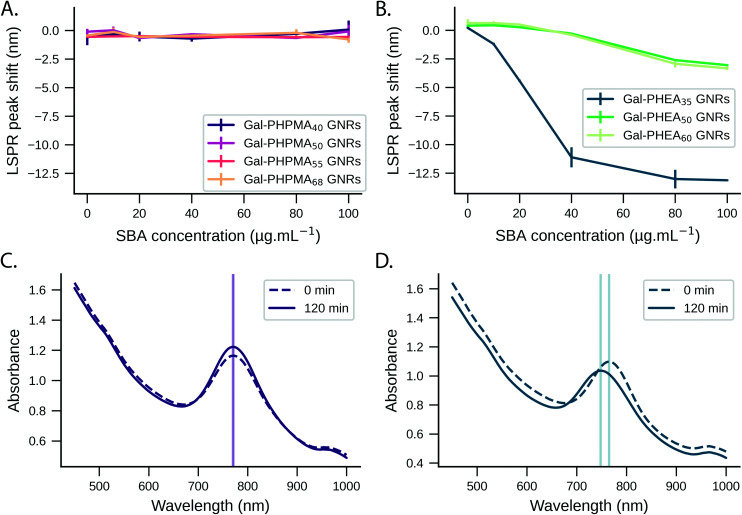
Response of nanoparticles to lectin in serum. LSPR peak shift of Gal-PHPMA GNRs (A) and Gal-PHEA GNRs (B) in serum as a function of SBA concentration and measured after 2 hours of incubation. LSPR wavelength peak shift is expressed relative to the peak position at the start of the injection (*N* = 3, mean ± SD). Representative UV-Vis spectra of Gal-PHPMA_40_ (C) and Gal-PHEA_35_ (D) coated GNRs in serum at time zero and 2 hours after addition of 100 μg mL^−1^ SBA are shown.

We monitored the LSPR peak following the addition of glyco-GNRs to a dilution series of SBA in serum. The Gal-PHPMA GNRs (DP 40, 50, 55, 68) showed no LSPR shift in the concentration range tested (Fig. 3A and C and control trials S14A/B†), in contrast to the strong LSPR shift observed in buffer alone. In comparison, Gal-PHEA nanorods showed dose-dependent LSPR shifts in serum plus SBA (Fig. 3B), which is the opposite to what is seen for PHPMA. While aggregation normally leads to a broadening of the LSPR peak, here the shape of the LSPR peak of Gal-PHEA GNRs in serum did not change significantly (Fig. 3D) and hence aggregation can be excluded. This is in contrast to the aggregative behaviour observed for the same assay performed in buffer, and confirms that initial screening in buffer is not be a reliable strategy for identification of the best-performing surface modifications for biosensing in blood/serum samples.

**Fig. 4:**
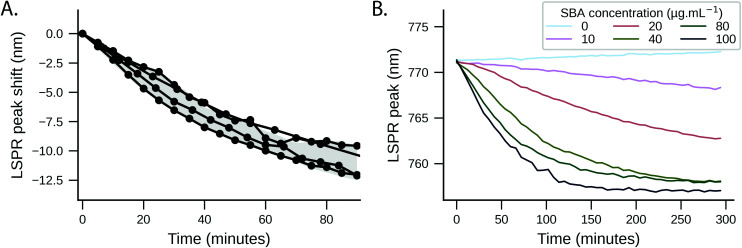
SBA (100 μg mL^−1^) binding to Gal-PHEA_35_ coated GNRs in serum. (A) LSPR peak shift Gal-PHEA_35_ GNRs with 100 μg mL^−1^ SBA in serum over time (*N* = 4). The shaded range represents the standard deviation of the data. (B) Evolution of LSPR peak wavelength location over time (5 hours) after addition of Gal-PHEA_35_ GNRs to serum containing different SBA concentrations.

The response of Gal-PHEA_35_ GNR to SBA in serum was found to be reproducible between batches of GNRs and polymer (Fig. 4A) confirming this conflicting behaviour (compared to what is seen in buffer alone) was a real effect. We also assayed the response kinetics of Gal-PHEA_35_ GNR over 5 hours using different SBA concentrations (Fig. 4B). In further control experiments, PHEA GNRs without glycan modification exhibited excellent stability and minimal response to SBA injection (Fig. S14C†), while Gal-PHEA GNRs showed no response upon addition of WGA (Fig. S14D†), confirming the selective SBA recognition of Gal-PHEA GNRs in complex serum conditions.

### Impact of serum proteins on sensing performance

From the above studies, the most significant observation was that in the case of Gal-PHEA GNRs in serum, the LSPR peak wavelength decreases upon SBA addition, consistent with overall loss of mass from the particle surface, rather than a net mass gain due to lectin binding, which would have been expected if a lectin was being captured by the rods in buffer. Secondly, the PHPMA coated GNRs showed no response to SBA in serum despite showing LSPR shifts in buffer. To explore the biocorona formation and the impact this had during SBA biosensing, we used differential centrifugal sedimentation (DCS). To estimate the biocorona thickness from the DCS data we used the core–shell model of Monopoli *et al.*^60^ where particles are treated as a high-density metallic core with a lower-density shell of biomolecules. If the size and density of the core nanoparticle are known and the density of the corona can be estimated, then the shell thickness can be calculated from the shift in particle mobility before and after corona formation.

**Fig. 5:**
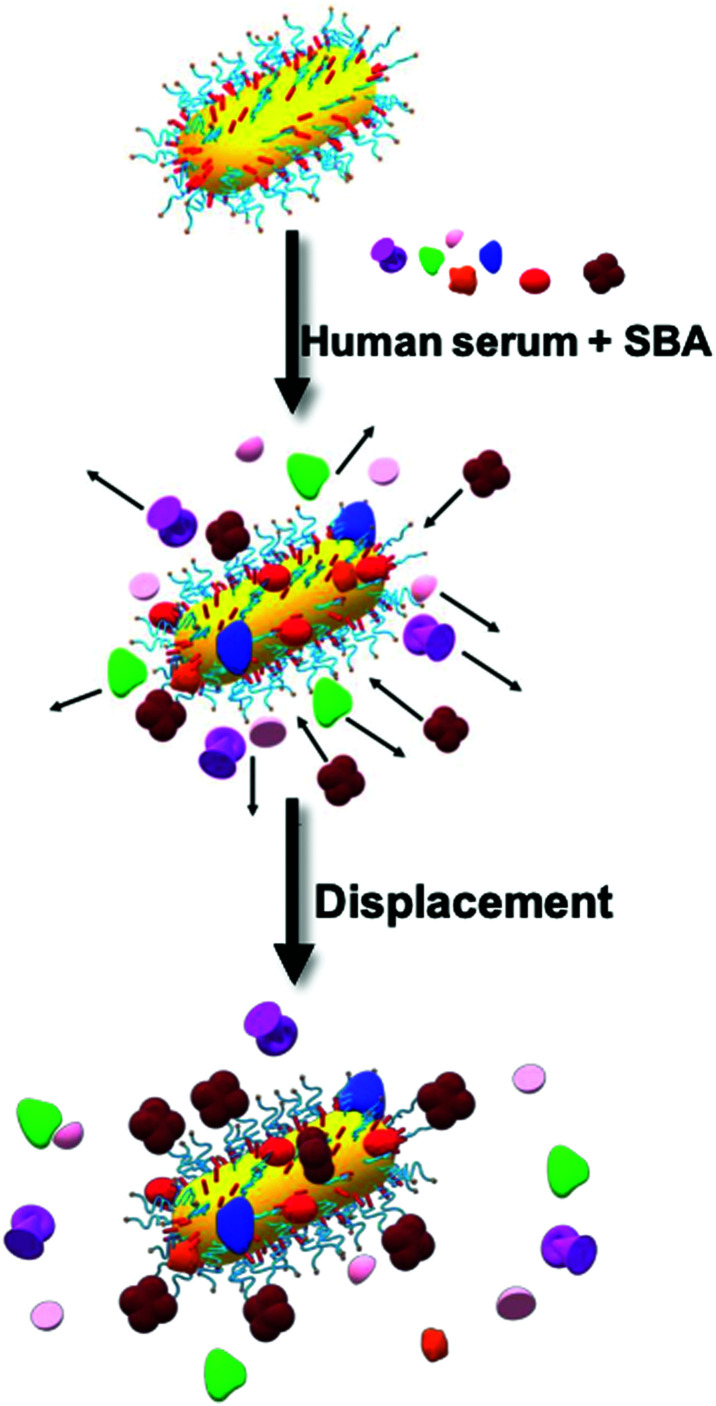
SBA binding to Gal-PHEA GNRs in serum. Schematic illustration of the formation of a biocorona surrounding Gal-PHEA coated GNR in a complex biological environment. In serum spiked with SBA, we hypothesise a displacement of the serum-derived biocorona due to glycan–lectin binding.

We observed an increase in coating thickness upon incubation in serum for both Gal-PHPMA_40_ and Gal-PHEA_35_ GNRs (Table 2 and Fig. S15 and S16†). The corona thickness was higher for Gal-PHPMA_40_ rods (3.3 ± 0.2 nm) as compared to Gal-PHEA_35_ GNRs (2.2 ± 0.2 nm). Upon addition of SBA to Gal-PHPMA_40_ GNRs there was no change in the thickness of the corona. However, in the case of Gal-PHEA_35_ there was actually a decrease in total corona thickness following addition of SBA, suggesting that the underlying mechanism for the Gal-PHEA GNRs response in media could be due to a coronal displacement mechanism, which does not occur for Gal-PHPMA GNRs. Coronal displacement has been exploited by Rotello *et al.*^70,71^ whereby a fluorescence polymer non-specifically bound to gold particle surfaces is displaced to generate signal. Fig. 5 proposes a mechanism for the sensing seen here, whereby in the case of Gal-PHEA with a relatively thin corona, addition of SBA leads to binding and displacement of weakly-bound biocorona components, leading to the red shift (shorter wavelength) of the LSPR. It is known that the behaviour of nanoparticles in complex biological matrices is a highly dynamic process and that the rates of exchange are governed by the nanoparticle surface properties,^53,72^ supporting this hypothesis. The different response of PHPMA GNRs can be attributable to its lower grafting density (compared to PHEA) and hence more vacancies on the GNR for formation of a ‘hard’ non-reversible corona, supported by the DCS data. The PHPMA also has fewer Gal residues and hence a likely decreased affinity for SBA may also contribute. It should be noted that these experiments do not rule out all SBA binding, just the lack of optical responses or significant corona displacement.

**Table 2:** Differential centrifugal sedimentation analysis

Sample	Medium	Peak size (nm)	Calculated coating thickness (nm)
PHEA_35_ GNRs	Buffer	19.7 ± 0.1	1.5 ± 0.1
	Serum	18.7 ± 0.1	2.2 ± 0.2
	Serum + SBA	18.1 ± 0.1	2.8 ± 0.2
Gal-PHEA_35_ GNRs	Buffer	19.7 ± 0.1	1.5 ± 0.1
	Serum	18.7 ± 0.1	2.2 ± 0.2
	Serum + SBA	19.5 ± 0.1	1.6 ± 0.1
PHPMA_40_ GNRs	Buffer	19.7 ± 0.2	1.5 ± 0.1
	Serum	17.6 ± 0.1	3.3 ± 0.2
	Serum + SBA	17.5 ± 0.2	3.4 ± 0.2
Gal-PHPMA_40_ GNRs	Buffer	19.8 ± 0.1	1.4 ± 0.1
	Serum	17.6 ± 0.1	3.3 ± 0.2
	Serum + SBA	17.4 ± 0.1	3.5 ± 0.2

**Fig. 6:**
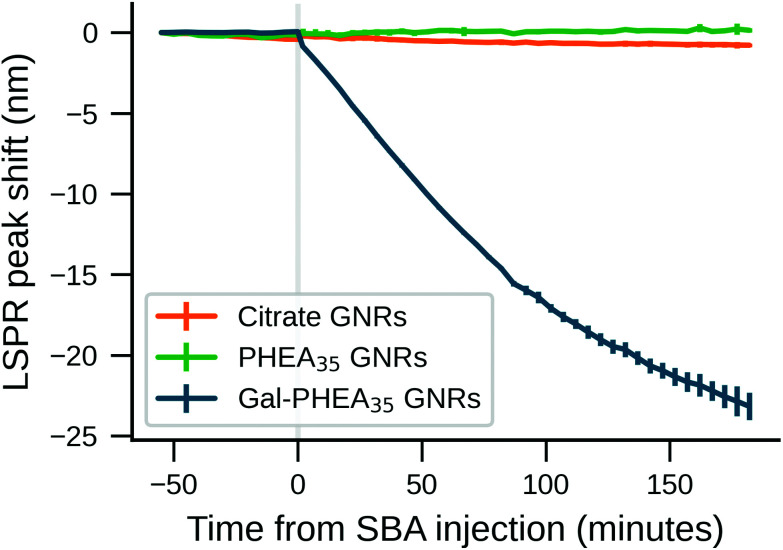
LSPR peak shift (nm) over time for citrate-GNRs, PHEA_35_ GNRs and Gal-PHEA_35_ GNRs. The samples were incubated in serum to allow the formation of a biocorona, followed by washing the rods with buffer. Absorbance spectra were monitored for 1 hour in buffer, before spiking (time zero) with SBA at 100 μg mL^−1^ (*N* = 3, mean ± SD).

To further explore the role of biocorona on the observed sensing outputs bare citrate-GNRs, PHEA_35_ GNRs and Gal-PHEA_35_ GNRs were incubated in human serum to allow corona formation. After this, the rods were repeatedly washed with buffer, to remove the most weakly bound coronal components. This treatment leaves behind a “hard” corona composed of biological molecules that have the highest affinity for the particle surface.^53,73^ Gal-PHEA_35_ GNRs prepared in this way exhibited a pronounced blue-shift of the LSPR peak upon the addition of 100 μg mL^−1^ SBA in buffer (Fig. 6), which was larger than seen without washing, (Fig. 4) presumably due to the absence of competing serum proteins in solution and increased glycan accessibility. Nanoparticle tracking analysis confirmed a decrease of the apparent particle size (Table 3 and Fig. S17†) upon addition of SBA and hence supported the hypothesis that SBA binding can displace some of the weaker bond serum proteins upon glycan binding. Non-glycosylated PHEA GNRs treated in the same manner showed no change in particle size using NTA upon SBA addition confirming that the displacement is due to specific lectin/glycan interactions. This data also shows that the biocorona does not completely shield the glycans on the GNR surface and that there is a dynamic exchange between hard-corona proteins and SBA (see illustration of proposed scheme in Fig. S18†). Moreover, the aspect ratio of the GNRs (3.8) employed in this study corresponds to the long GNRs (3.7) studied by Ferhan *et al.*, which were observed to show superior plasmonic surface sensitivity compared to short GNR and long GNRs deposited on sensor substrate.^27^

**Table 3:** Nanoparticle tracking analysis particles in buffer and serum

Sample	Medium	Mode (nm)
Citrate-GNRs	Water	43 ± 1.6
PHEA_35_ GNRs	Buffer	54.5 ± 2.8
	Serum, then buffer	98.5 ± 4.4
	Serum, then buffer + SBA	100.8 ± 1.5
Gal-PHEA_35_ GNRs	Buffer	52.6 ± 1.5
	Serum, then buffer	106.1 ± 4.9
	Serum, then buffer + SBA	61.3 ± 5.7

## Conclusions

Here we demonstrate how the nature of polymeric tethers, used to anchor glycans to gold nanorods, directs the formation of biocoronas in serum, and the impact this has on biosensing of lectins. It is shown that prevention of biocorona formation may not be essential, and that a soft-corona is not only tolerated, but its displacement upon lectin binding actually leads to signal generation. In contrast a ‘hard’ irreversible corona effectively prevented biosensing. In simple buffer solutions, glycosylated poly(*N*-hydroxypropyl methacrylamide)-coated nanorods, showed a dose-dependent LSPR red-shift upon addition of soybean agglutinin (SBA). However, in serum, which contains a large range of biological macromolecules, no signal was detected which is attributed to biocorona formation. In contrast, glycosylated poly(*N*-hydroxyethyl acrylamide)-coated nanorods showed very different behaviour including a lectin dose dependant blue-shift in serum. Using a combination of UV-Visible spectroscopy, differential centrifugation sedimentation and nanoparticle tracking analysis the underlying mechanism for these behaviours was proposed and harnessed to enable biosensing of lectins in serum. Poly(*N*-hydroxypropyl methacrylamide)-coated nanorods were observed to have lower polymer grafting densities, which upon incubation with serum led to the formation of a thick and irreversibly bound ‘hard’ protein corona and hence no lectin binding was observable. Changing the polymeric coating to poly(*N*-hydroxyethyl acrylamide) also resulted in biocorona formation in serum, but this was a ‘soft’ corona containing irreversibly bound components which could be displaced by addition of lectin. This displacement mechanism leads to the blue-shifted lectin biosensing outputs (overall loss of mass) in serum compared to red-shifted (gain in mass) which is seen in buffer. The role of grafting density in biocorona formation, and lectin binding responses, was supported by the use of different polymer chain-lengths on GNRs, with the shortest polymer coatings (whilst still being long enough to provide colloidal stability) giving the strongest responses, consistent with a grafting-density dependant process. These results illustrate how careful selection of the polymer coating on gold nanorods is an accessible and powerful tool to control observable biosensing responses. It also shows that preventing all biocorona formation, which is incredibly challenging, on plasmonic nanoparticle sensors may not be essential, so long as the corona which does form is reversible and the underlying targeting ligands remain exposed. Finally, this work clearly shows that the polymer coating, not just the targeting ligand, plays a critical role in tuning the biosensing outputs and that the coating must be tuned for each application area.

## Conflicts of interest

There are no conflicts to declare.

## Supplementary Material

NR-013-D1NR01548F-s001
