# Supplementary material for: The polymeric glyco-linker controls the signal outputs for plasmonic gold nanorod biosensors due to biocorona formation
Source: Nanoscale. 2021 Jun 3;13(24):10837–48. doi: 10.1039/d1nr01548f (PMC8223873; doi:10.1039/d1nr01548f)
Supplement: NR-013-D1NR01548F-s001 [file NR-013-D1NR01548F-s001.pdf]

Electronic Supplementary Material (ESI) for Nanoscale.
This journal is © The Royal Society of Chemistry 2021

Electronic Supplementary Information

"The Polymeric Glyco-Linker Controls the Signal Outputs for Plasmonic Gold Nanorods Biosensors due to Biocorona Formation"

Alessia Pancaro,[a,b] Michal Szymonik,[a] Panagiotis G. Georgiou,[c] Alexander N. Baker,[c] Marc Walker,[e] Peter Adriaensens,[f] Jelle Hendrix,[b] Matthew I. Gibson[c,d] and Inge Nelissen*[a]*

[a] Health Unit, Flemish Institute for Technological Research (VITO), Boeretang 200, Mol, BE-2400, Belgium.

[b] Dynamic Bioimaging Lab, Advanced Optical Microscopy Centre and Biomedical Research Institute, Hasselt University, Agoralaan C, Diepenbeek, BE-3590, Belgium.

[c] Department of Chemistry, University of Warwick, Gibbet Hill Road, Coventry, CV4 7AL, UK.

[d] Warwick Medical School, University of Warwick, Gibbet Hill Road, Coventry, CV4 7AL, UK.

[e] Department of Physics, University of Warwick, Gibbet Hill Road, Coventry, CV4 7AL UK.

[f] Applied and Analytical Chemistry, Institute for Materials Research, Hasselt University, Agoralaan D, Diepenbeek, BE-3590, Belgium.

*<u>*Corresponding Authors:*</u> inge.nelissen@vito.be and m.i.gibson@warwick.ac.uk

EXPERIMENTAL

Synthetic Methods

Photo-polymerisation of *N*-(2-hydroxypropyl)methacrylamide (HPMA) *via* photo-initiated RAFT. The following procedure describes a reaction for [monomer]:[PFP-DMP] ratio of 40. In a typical reaction, *N*-(2-hydroxypropyl)methacrylamide (HPMA) (0.43 g, 3.01 mmol) and RAFT agent of 2-(dodecylthiocarbonothiolythio)-2-methylpropanoic acid pentafluorophenyl ester (PFP-DMP) (0.04 g, 0.075 mmol) were dissolved in 1 : 3 dioxane : methanol solution (2 mL) in a vial. The resulting solution was degassed by sparging N_2(g) for 15 min and the sealed vial was incubated at 37 ˚C with magnetic stirring under 460 nm light irradiation for 120 min. The reaction was rapidly cooled and precipitated twice into diethyl ether to yield a yellow polymer product which was further dried under vacuum. An aliquot of crude polymerisation mixture was taken for ^{1}H NMR in methanol-d_4 for conversion and $M_{n,}$ NMR analysis. ^{1}H, ^{19}F NMR and SEC analysis was finally conducted on purified polymer after precipitation. The same procedure was followed for [HPMA]:[PFP-DMP CTA] ratios of 60, 80, 100 and 120. Conversions were calculated using ^{1}H NMR spectroscopy by comparing the integrations of the HPMA monomer signals (δ 5.73 ppm) with those of the corresponding signals of the polymer (δ 1.31-1.04 ppm. C$\boldsymbol{H_3}$ of PHPMA backbone and C$\boldsymbol{H_3}$ of PHPMA side chain). $M_{n.\,NMR}$ was calculated by end-group analysis, i.e., by comparing the integrations of the -CH_3 signals (δ 0.92 ppm) of dodecyl end group with those of the corresponding methyl signals of the polymer (δ 1.31-1.04 ppm). ^{1}H NMR (400 MHz. methanol-d_4): δ (ppm) 7.53 (br m, N***H*** of PHPMA side chain), 3.88 (br s, C***H*** of PHPMA side chain), 3.19- 3.02 (br m, C$\boldsymbol{H_2}$ of PHPMA sidechain), 2.05−1.79 (br m, C$\boldsymbol{H_2}$ of PHPMA backbone), 1.31−1.04 (br m, C$\boldsymbol{H_3}$ of PHPMA backbone and C$\boldsymbol{H_3}$ of PHPMA side chain), 0.92 (t, 3H, CH_2-CH_2-C$\boldsymbol{H_3}$ of dodecyl end-group). FT-IR (neat): v (cm^{-1}) 3300 (N-H and O-H stretch); 2920 (alkyl C-H stretch); 1775 (C_6F_5C=O stretch); 1630 (amide C=O stretch); 1518 (N-H bend); 1443 (C-H bend); 1200 (C-O stretch); 1080 (C-O stretch); 993 (C-F stretch).

Photo-polymerisation of *N*-(2-hydroxyethyl) acrylamide (HEA) *via* photo-initiated RAFT. The following procedure describes a reaction for [monomer]:[PFP-DMP] ratio of 120. *N*-(2-hydroxyethyl) acrylamide (HEA) (1.30 g, 11.3 mmol) and raft agent of 2-(dodecylthiocarbonothiolythio)-2-methylpropanoic acid pentafluorophenyl ester (PFP-DMP) (0.05 g, 0.094 mmol) were dissolved in 50:50 dioxane:methanol solution (5.41 mL) in a vial.

The resulting solution was degassed by sparging N_2(g) for 15 min and the sealed vial was incubated at 37 ˚C with magnetic stirring under 460 nm light irradiation for 120 min. The reaction was rapidly cooled and precipitated into diethyl ether. The polymer was reprecipitated into diethyl ether from methanol twice to yield a yellow polymer product which was further dried under vacuum. An aliquot of crude polymerisation mixture was taken for ^{1}H NMR in methanol-d_4 for conversion and $M_{n, NMR}$ analysis. ^{1}H, ^{19}F NMR and SEC analysis was finally conducted on purified polymer after precipitation. Same procedure was followed for [HEA]:[PFP-DMP] ratios of 140, 160, 180 and 200. Conversions were calculated using ^{1}H NMR spectroscopy by comparing the integrations of the HEA monomer signal (δ 5.67 ppm) with this of the C***H*** of the PHEA backbone (δ 2.22-2.04 ppm). $M_{n. NMR}$ was calculated by end-group analysis, i.e., by comparing the integration of the -CH_3 signal (δ 0.92 ppm) of the dodecyl end-group with this of the C***H*** of the PHEA backbone (δ 2.22-2.04 ppm). ^{1}H NMR (400 MHz. methanol-d_4): δ (ppm) 8.15-8.03 (br m, N***H*** of PHEA side chain), 3.89-3.13 (br m, NH-C$\boldsymbol{H_2}$ and C$\boldsymbol{H_2}$-OH of PHEA side chain), 2.35−2.05 (br m, C***H*** of PHEA backbone), 1.85-1.31 (br m, C$\boldsymbol{H_2}$ of PHEA backbone), 0.92 (t, 3H, CH_2-C$\boldsymbol{H_3}$ of dodecyl end-group). FT-IR (neat): v (cm^{-1}) 3300 (N-H and O-H stretch); 2868 (alkyl C-H stretch); 1772 (C_6F_5C=O stretch); 1638 (amide C=O stretch); 1544 (N-H bend); 1438 (C-H bend); 1216 (C-O stretch); 1060 (C-O stretch); 950 (C-F peak on shoulder of 1060 peak).

End-group modification of PFP-poly(*N*-(2-hydroxypropyl) methacrylamide) (PFP-PHPMA) and PFP-poly(*N*-hydroxyethyl acrylamide) (PFP-PHEA) homopolymers using galactosamine.

In a typical reaction, $PFP-PHPMA_{40}$ (100 mg, 0.011 mmol), galactosamine (11.4 mg, 0.053 mmol) were dissolved in 5 mL DMF with 0.05 M triethylamine (TEA) (50 µL). The reaction was stirred at 50 ˚C for 16 hrs. The polymer was precipitated into diethyl ether from methanol three times and dried over under vacuum. ^{19}F-NMR and IR analysis were performed and confirmed the loss of the pentafluoro end-group.

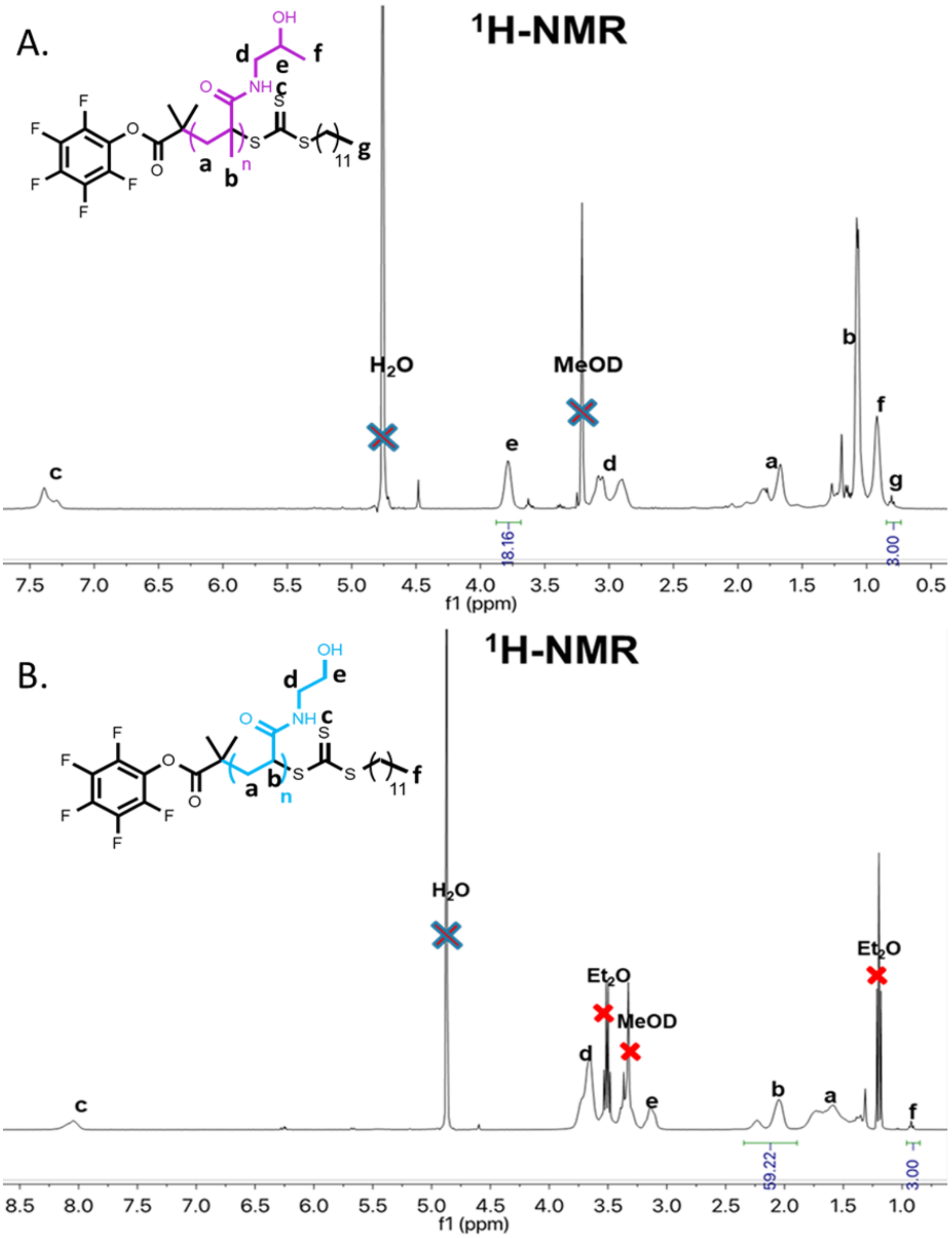

Figure S1. ^{1}H-NMR spectra of PFP-PHPMA$_{40}$ (A) and PFP-PHEA$_{60}$ (B) recorded in methanol-*d*$_4$.

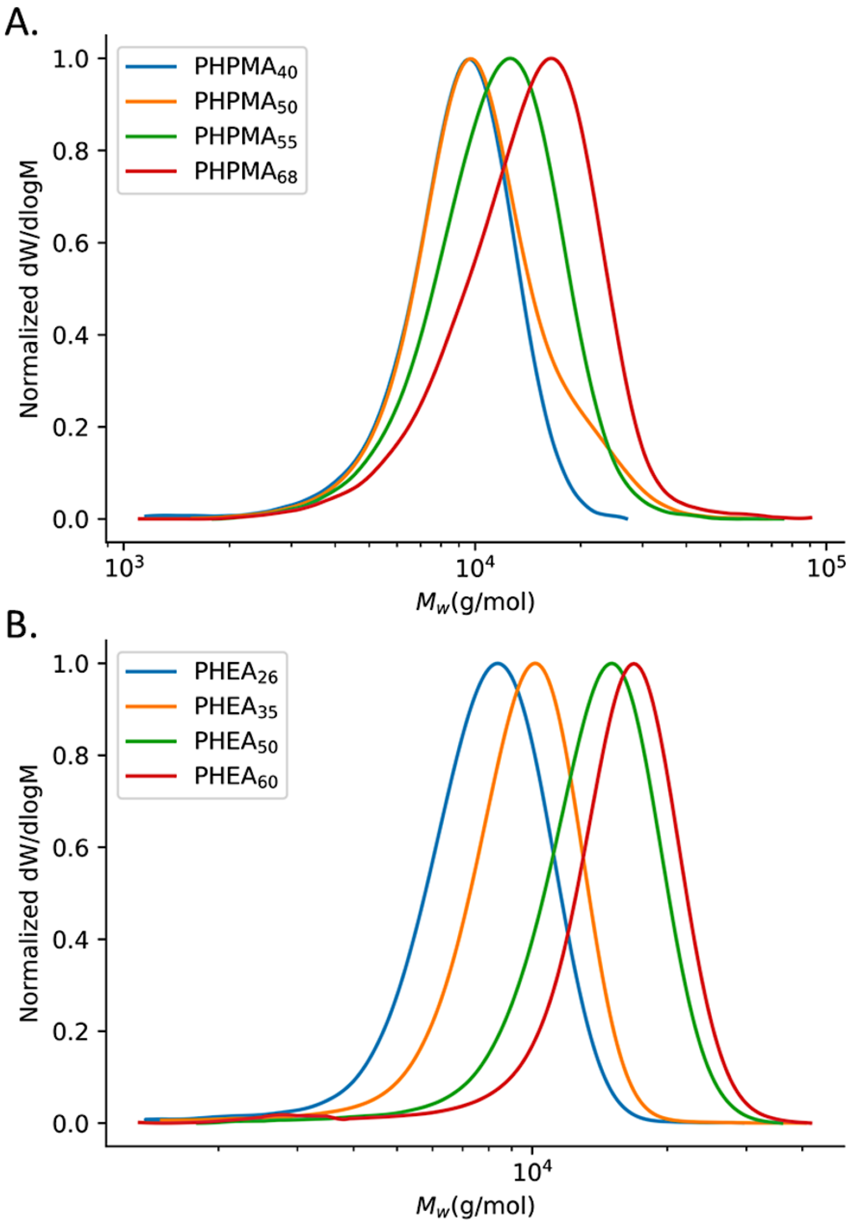

Figure S2. Normalised SEC RI (size exclusion chromatography - refractive index) molecular weight distributions for (A) PHPMA and (B) PHEA homopolymers using 5 mM NH_4BF_4 in DMF as the eluent.

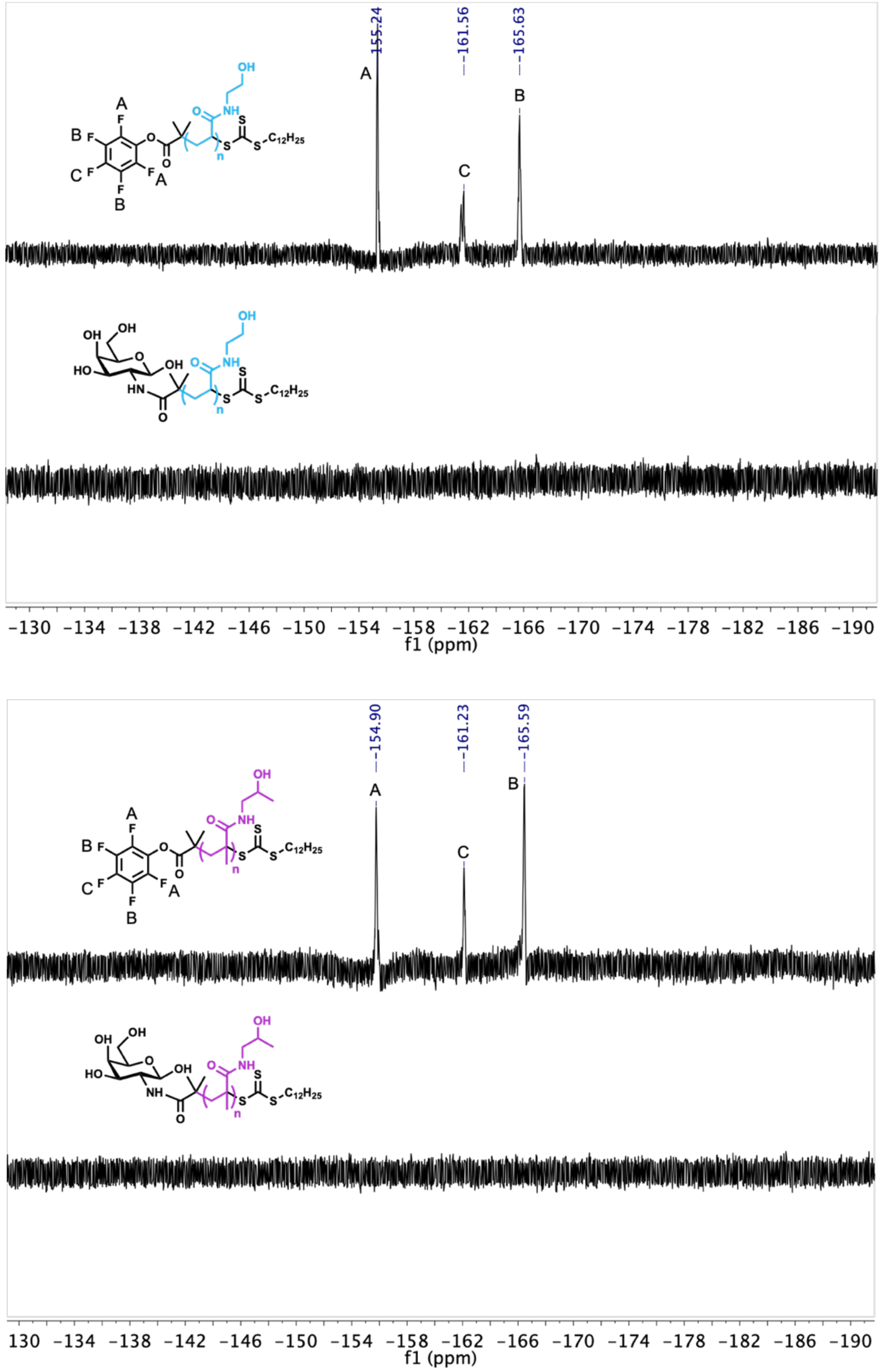

Figure S3. ^{19}F NMR spectra for the purified PFP/Gal-PHPMA$_{40}$ and PFP/Gal-PHEA$_{60}$ polymers before and after post-functionalisation with galactosamine. All spectra were recorded in methanol-d_4.

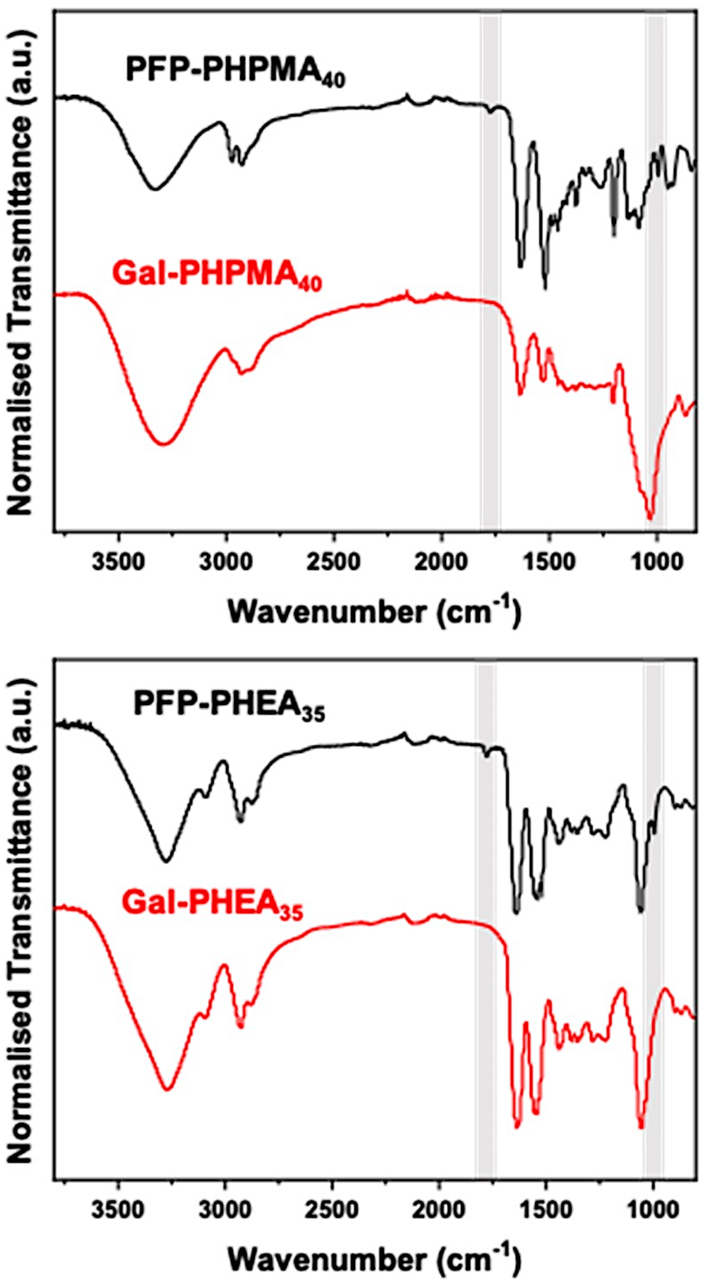

Figure S4. FT-IR spectra recorded for PHPMA and PHEA homopolymers. The samples were analysed before (black) and after (red) end-group modification with galactosamine. The disappearance of the characteristic vibration peaks of PFP group at 950 and 1750 cm^{-1} is shown.

Table S1. Glycopolymer-coated GNRs characterisation. UV-Vis LSPR peak (nm), ζ-potential (mV), and peak diameter (nm) by DCS and mode (nm) by NTA of citrate-GNRs and glycopolymer-coated GNRs.

A	UV-Vis LSPR peak (nm)	ζ-Potential (mV)	DCS Peak diameter (nm)	NTA size Mode (nm)
**Citrate-GNRs**	779 ± 1	- 44.9 ± 2.3	22.3 ± 0.1	43.1 ± 1.9
**Gal-PHPMA$_{40}$ GNRs**	788 ± 1	- 31.3 ± 1.6	19.8 ± 0.2	60.3 ± 1.5
**Gal-PHPMA$_{50}$ GNRs**	782 ± 1	- 33.9 ± 2.3	20.3 ± 0.1	62.4 ± 3.7
**Gal-PHPMA$_{55}$ GNRs**	783 ± 1	- 36.4 ± 1.8	20.2 ± 0.1	61.6 ± 1.1
**Gal-PHPMA$_{68}$ GNRs**	782 ± 1	- 35.1 ± 1.3	20.6 ± 0.1	64.0 ± 4.4
**Gal-PHEA$_{35}$ GNRs**	787 ± 1	- 33.8 ± 1.8	19.7 ± 0.1	62.3 ± 2.2
**Gal-PHEA$_{50}$ GNRs**	788 ± 2	- 37.1 ± 1.4	19.3 ± 0.1	63.9 ± 5.1
**Gal-PHEA$_{60}$ GNRs**	788 ± 2	- 33.7 ± 1.3	19.2 ± 0.1	62.8 ± 3.2

B	UV-Vis LSPR peak (Δnm)	ζ-Potential (ΔmV)	DCS Peak diameter (Δnm)	NTA size Mode (Δnm)
**Gal-PHPMA$_{40}$ GNRs**	10.3 ± 1.1	13.3 ± 0.4	2.6 ± 0.1	7.0 ± 0.1
**Gal-PHPMA$_{50}$ GNRs**	3.3 ± 0.5	11 ± 0.1	2.0 ± 0.1	8.6 ± 0.1
**Gal-PHPMA$_{55}$ GNRs**	4.5 ± 1.0	8.7 ± 0.4	2.1 ± 0.1	8.9 ± 1.0
**Gal-PHPMA$_{68}$ GNRs**	2.8 ± 0.3	10.4 ± 0.9	1.8 ± 0.2	11.8 ± 0.6
**Gal-PHEA$_{35}$ GNRs**	9.3 ± 1.2	10.5 ± 0.8	2.5 ± 0.2	9.5 ± 0.2
**Gal-PHEA$_{50}$ GNRs**	10.4 ± 1.3	7.9 ± 0.1	3.1 ± 0.1	10.9 ± 0.1
**Gal-PHEA$_{60}$ GNRs**	8.9 ± 0.9	10.6 ± 0.8	3.0 ± 0.1	9.5 ± 0.5

A. After glycopolymer functionalisation, UV-Vis LSPR peak showed a red shift, ζ-potential decrease, while NTA and DCS indicated an increased in particle size, confirming the successful attachment of the glycopolymers to the particle surface. Table A shows the mean ± SD of two technical replicates of UV-Vis, ζ-potential, DCS and NTA measurements for one representative batch of GNRs, respectively.

B. Since batch-to-batch variation of LSPR peak are common, average changes in the above parameters of different batches were calculated of citrate-GNRs and the corresponding glycopolymer-coated GNRs (Table B). We performed N = 4 (UV-Vis); N = 3 (ζ-potential); N = 3 (DCS); N = 2 (NTA).

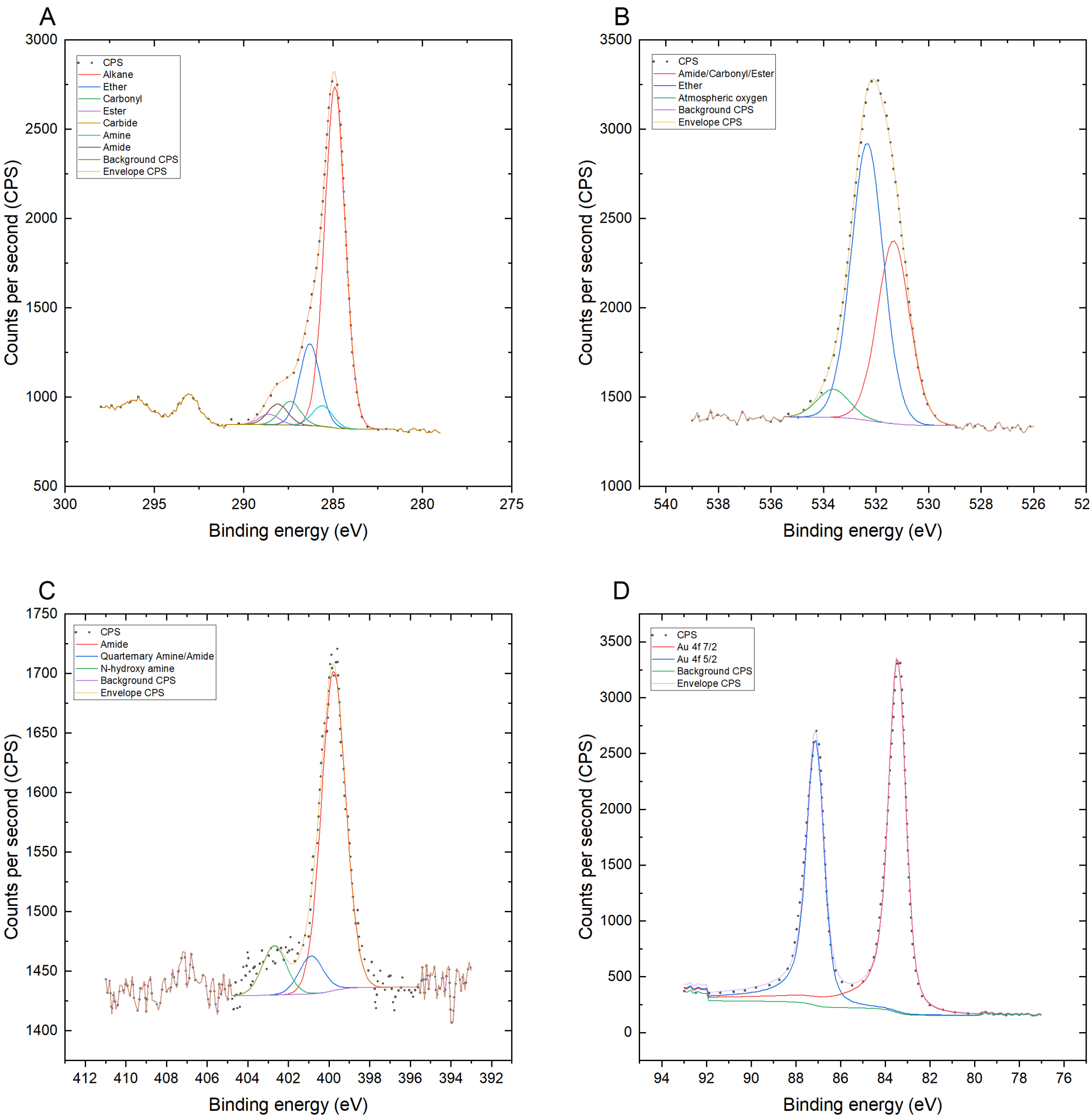

Figure S5. XPS scans of Gal-PHPMA$_{40}$ GNRs A) C 1*s* B) O 1*s* C) N 1*s* D) Au 4*f*.

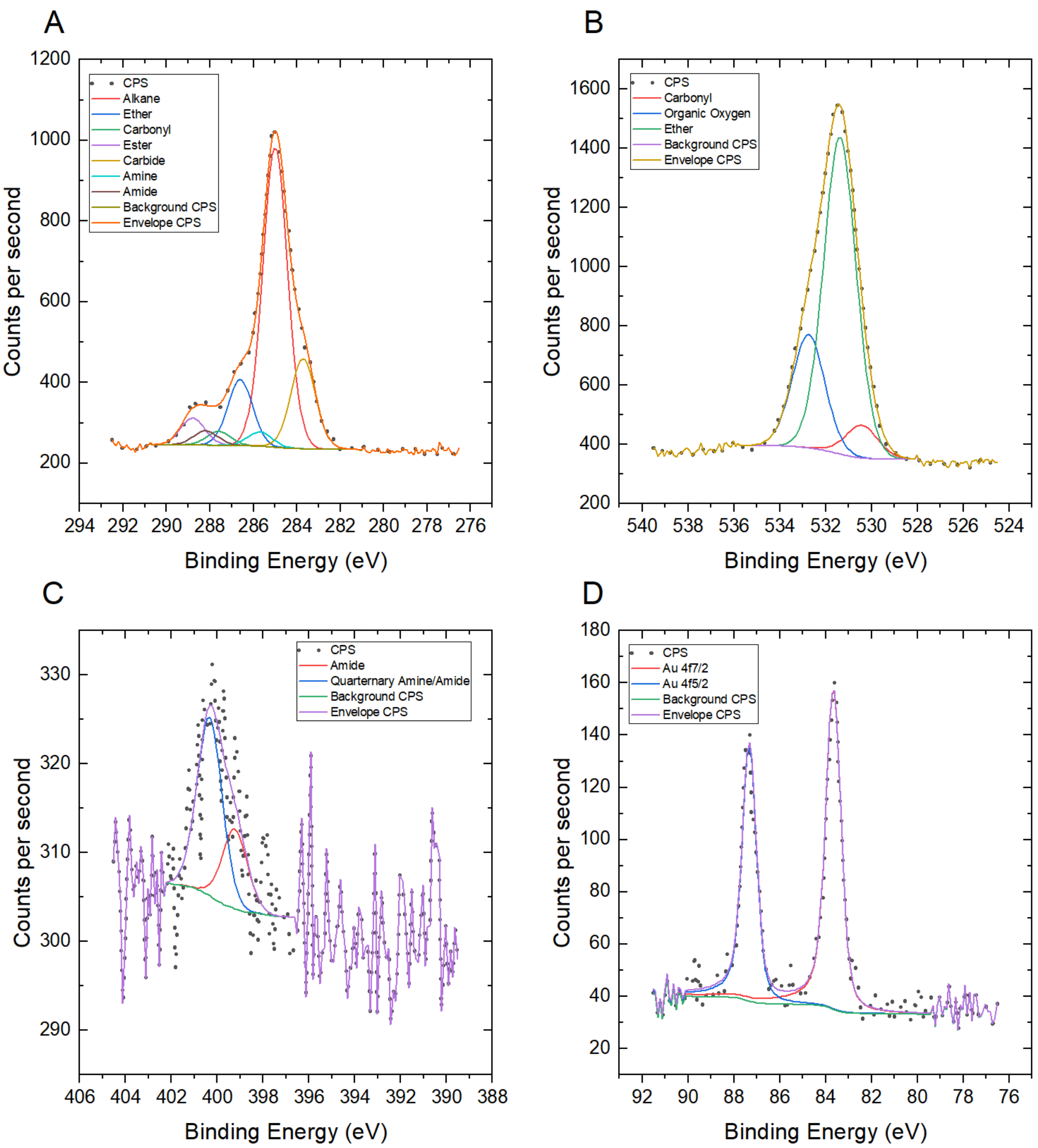

Figure S6. XPS scans of Gal-PHEA$_{35}$ GNRs A) C 1*s* B) O 1*s* C) N 1*s* D) Au 4*f*.

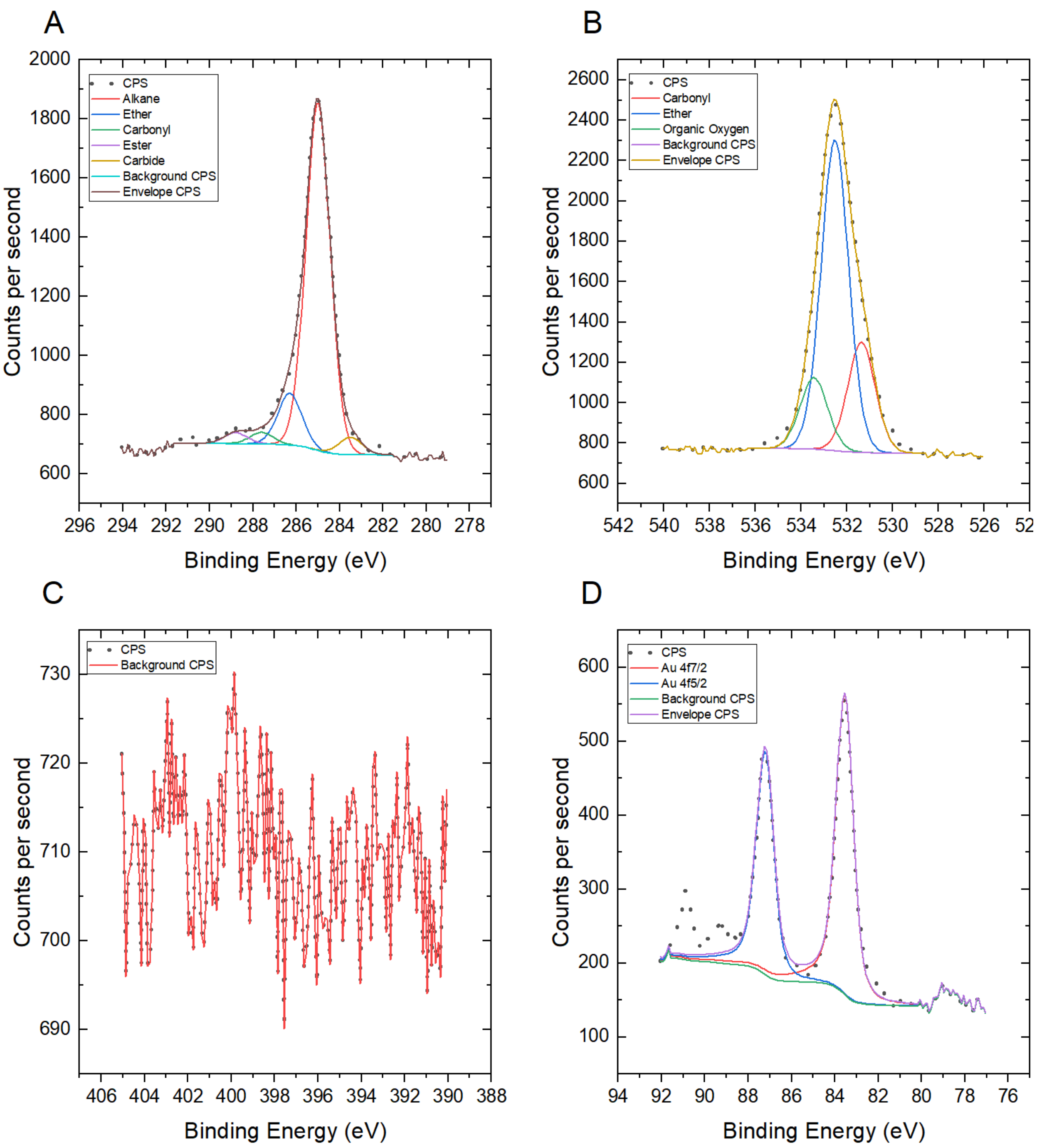

Figure S7. XPS scans of citrate-GNRs A) C 1*s* B) O 1*s* C) N 1*s* D) Au 4*f*.

Table S2. Elemental compositions of (Gal)-PHEA/PHPMA functionalised nanorods including N:Au ratios

Particle Composition			Elemental Percentage Composition (%)				Elemental Ratios	
**DP**	**Polymer**	**Sugar**	**C 1*s***	**O 1*s***	**N 1*s***	**Au 4*f***	**N 1*s*:C 1*s***	**N 1*s*:Au 4*f***
**0**	--------	(Citrate buffer)	57.27	41.41	0.00	1.32	0	0
**40**	PHPMA	No glycan	61.48	29.58	3.04	5.90	0.050	0.516
**35**	PHEA	No glycan	60.91	36.00	1.71	1.38	0.028	1.234
**40**	PHPMA	galactosamine	66.06	22.62	4.81	6.50	0.073	0.740
**55**	PHPMA	galactosamine	66.09	20.69	4.51	8.71	0.068	0.518
**35**	PHEA	galactosamine	62.82	35.99	0.81	0.38	0.013	2.147
**60**	PHEA	galactosamine	52.67	46.31	0.77	0.26	0.015	2.999

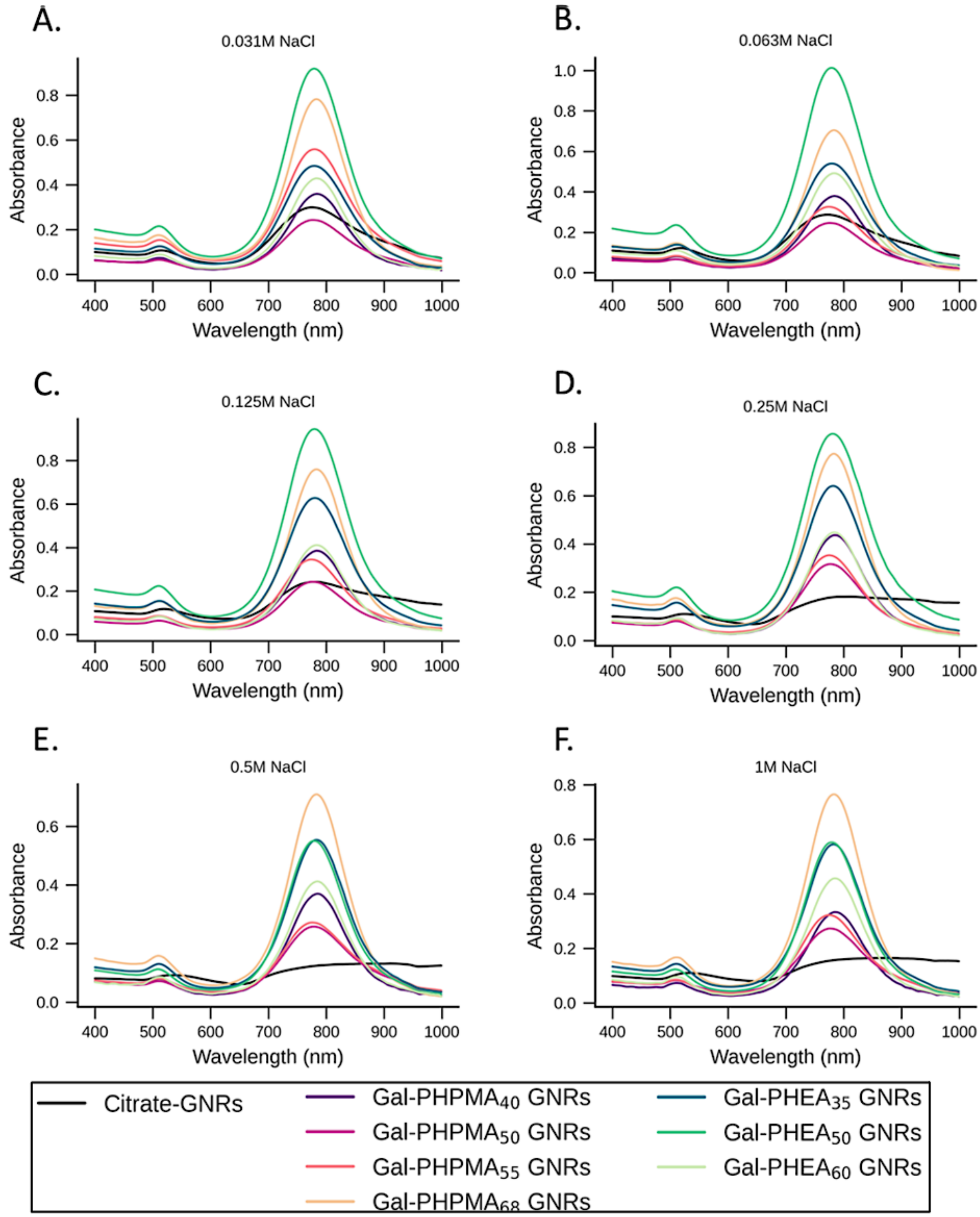

Figure S8. Salt titration assay. UV-Vis spectra of citrate-GNRs, Gal-PHPMA (DP 40, 50, 55, 68) and Gal-PHEA (DP 35, 50, 60)-coated GNRs upon incubation with different concentrations of NaCl: A = 0.031 M; B = 0.063 M; C = 0.125 M; D 0.25 M; E = 0.5 M; F = 1 M. A representative example out of three replicate measurements is shown.

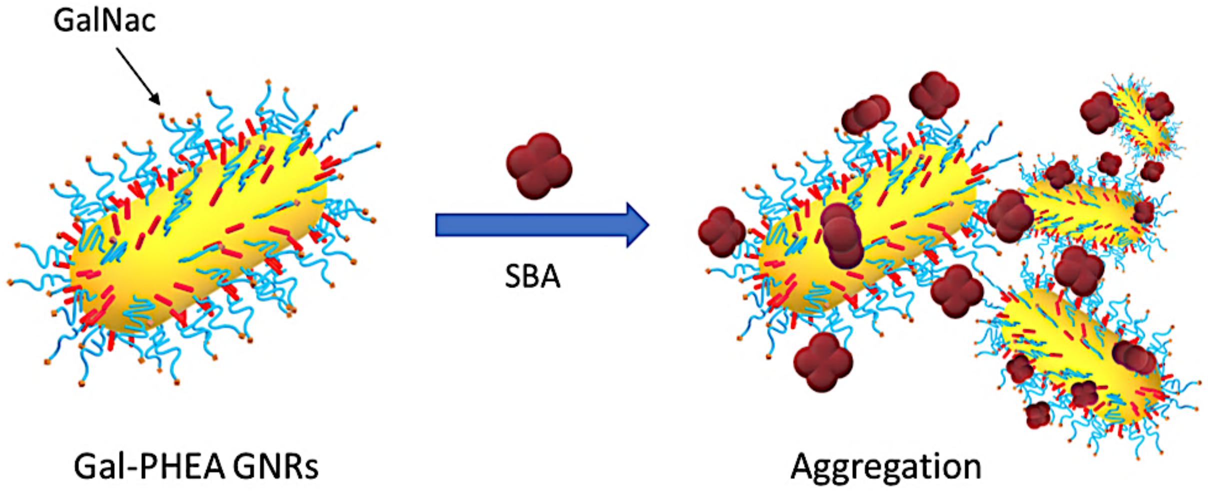

Figure S9. SBA binding event to Gal-PHEA GNRs in buffer. Schematic illustration for the recognition of Gal-PHEA coated GNRs by SBA in buffer, leading to GNRs aggregation.

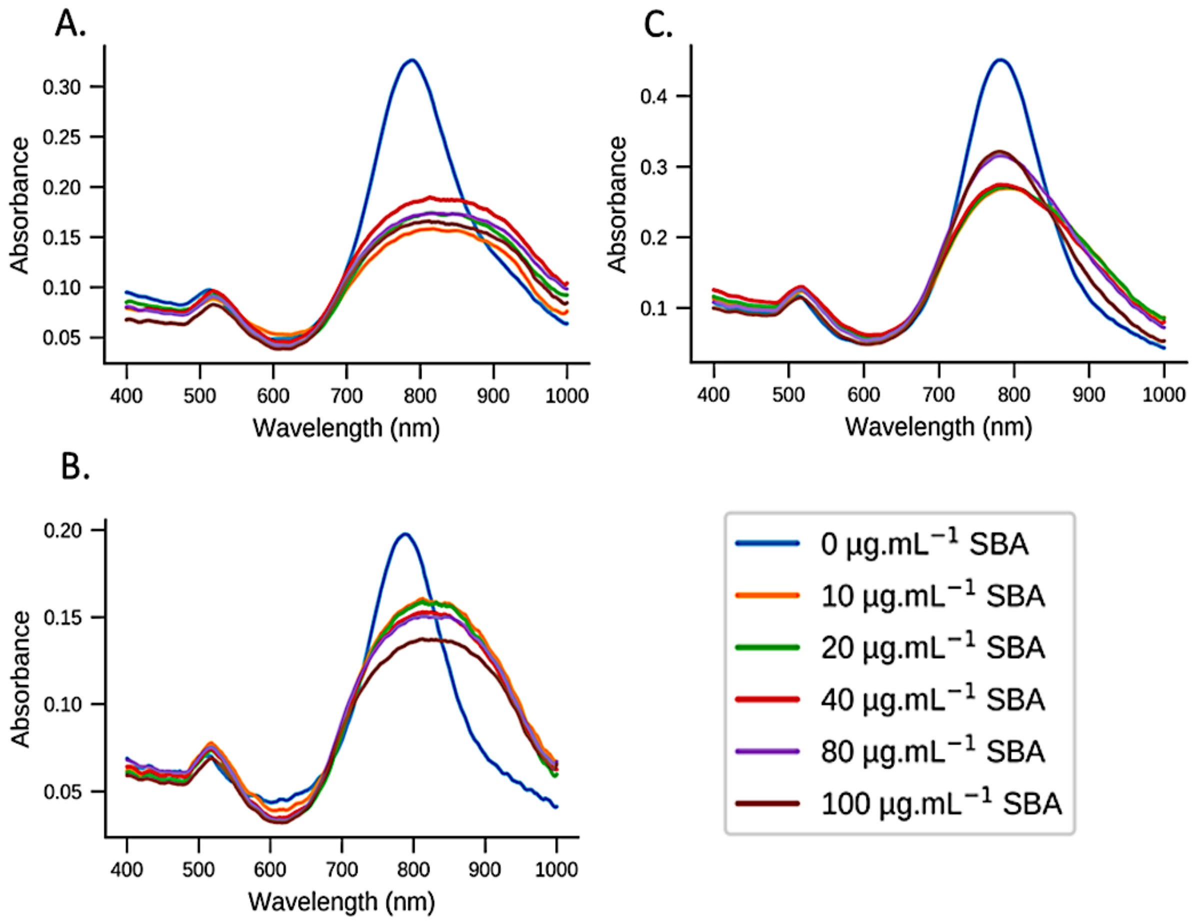

Figure S10. SBA binding in buffer to Gal-PHEA-coated GNRs with increasing linker length. UV-Vis spectra of Gal-$PHEA_{35}$ (A), Gal-$PHEA_{50}$ (B) and Gal-$PHEA_{60}$ (C) coated-GNRs after incubation in different concentrations of SBA in buffer medium for 2 hours. Shorter polymer linker lengths cause increasing instability and aggregation leading to a broadening of the LSPR peak. A representative example out of three replicate measurements is shown.

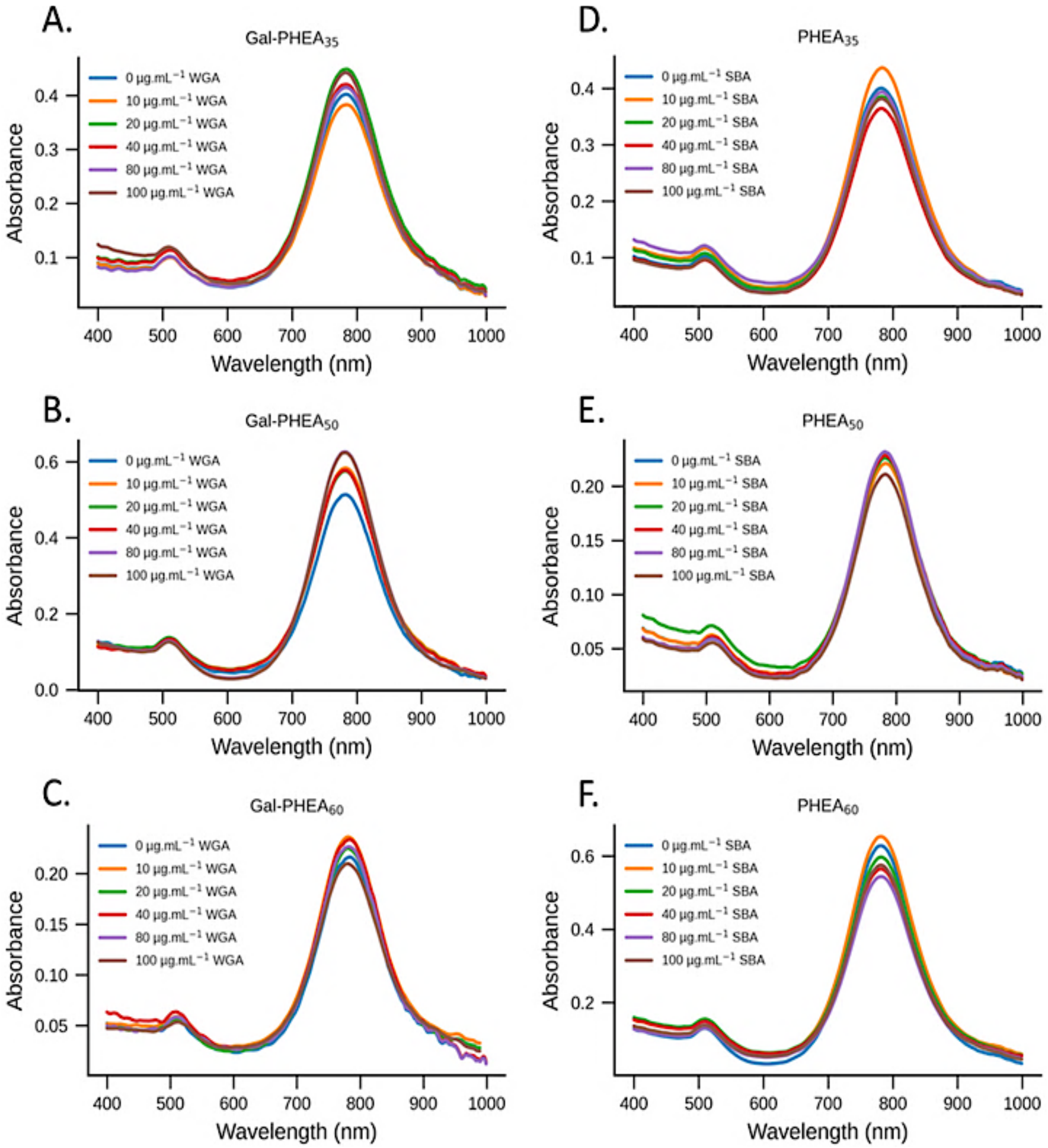

Figure S11. WGA binding in buffer to Gal-PHEA GNRs (left) and SBA binding in buffer to PHEA GNRs (right). UV-Visible spectra for Gal-PHEA$_{35}$ (A), Gal-PHEA$_{50}$ (B) and Gal-PHEA$_{60}$ (C) coated GNRs with different concentrations of WGA in buffer. UV-Visible spectra of PHEA$_{35}$ (D), PHEA$_{50}$ (E) and PHEA$_{60}$ (F) coated GNRs incubated with different concentrations of SBA in buffer for 2 hours. A representative example out of three replicate measurements is shown.

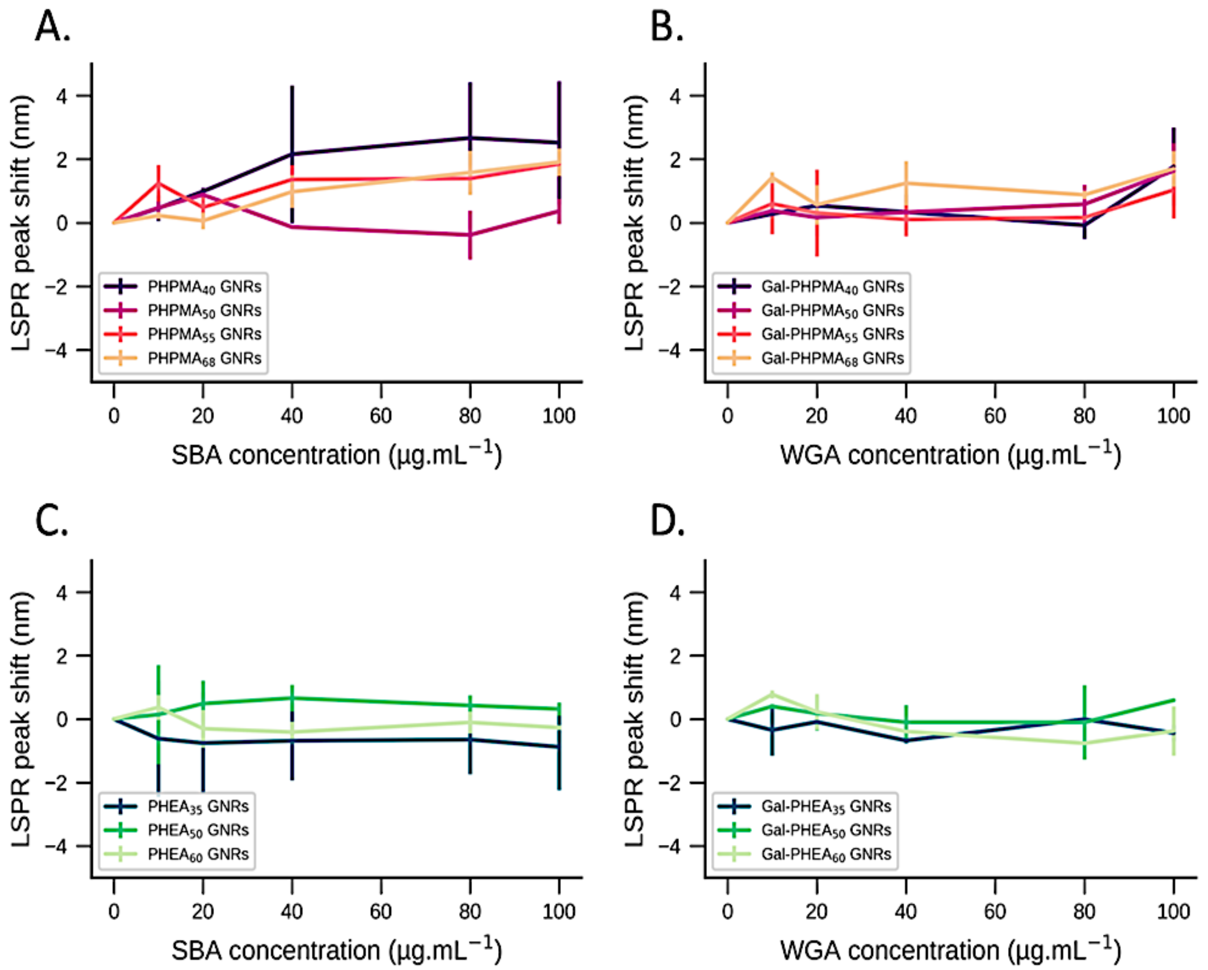

Figure S12. Control conditions for (Gal)-PHPMA and (Gal)-PHEA coated GNRs in buffer. LSPR peak shift of PHPMA (A) and PHEA (C) coated GNRs as a function of SBA concentration, determined by UV-Vis spectroscopy in buffer. Same analysis was performed for Gal-PHPMA (B) and Gal-PHEA (D) coated GNRs using different concentrations of WGA. All samples were analysed in duplicate every 30 mins for 2 hours (error bars: mean +/- SD after 2 hours).

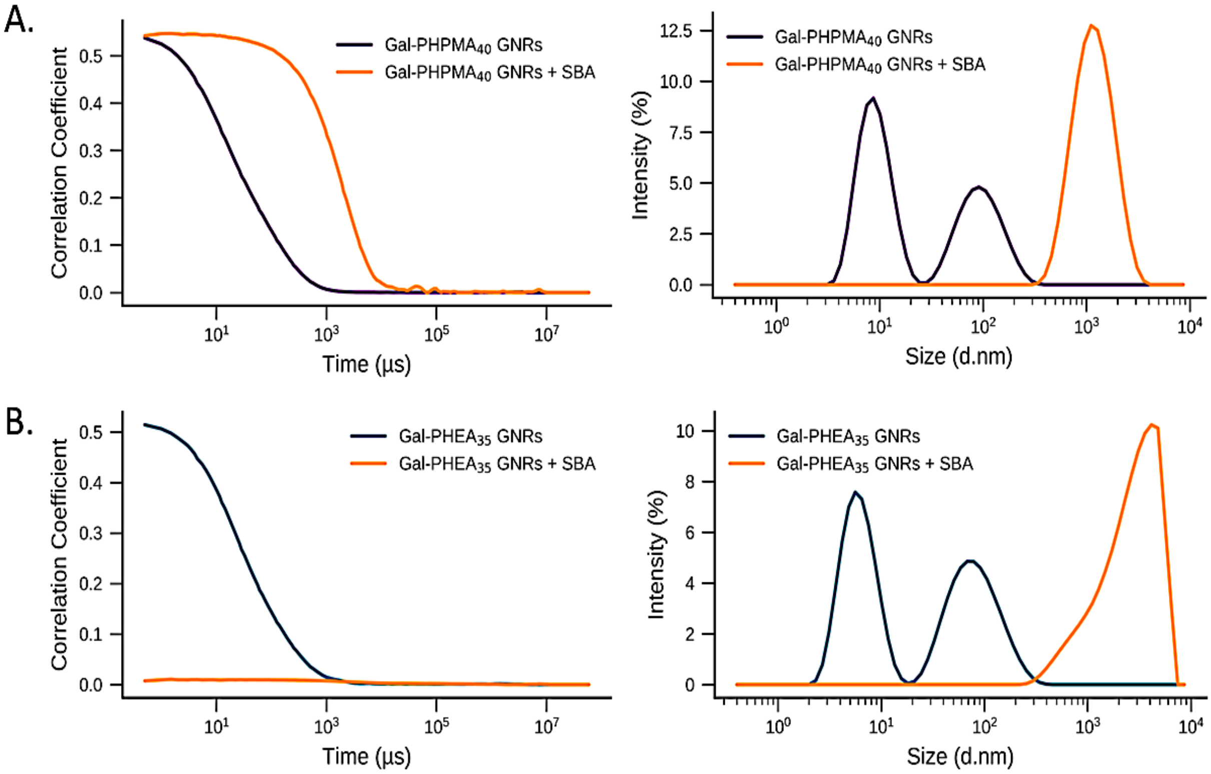

Figure S13. Dynamic light scattering analysis of Gal-PHPMA$_{40}$ and Gal-PHEA$_{35}$-conjugated GNRs before and after incubation with 100 µg.mL^{-1} of SBA in buffer at room temperature for 30 minutes. Autocorrelation functions (left) and intensity-weighted size distributions (right) plots for Gal-PHPMA$_{40}$ GNRs (A) and Gal-PHEA$_{35}$ GNRs (B). For Gal-PHPMA$_{40}$ GNRs, the correlation signal takes longer time to decay upon addition of SBA suggesting an increase in nanoparticle size consistent with binding, while the disappearance of the signal for Gal-PHEA$_{35}$ GNRs is consistent with aggregation.

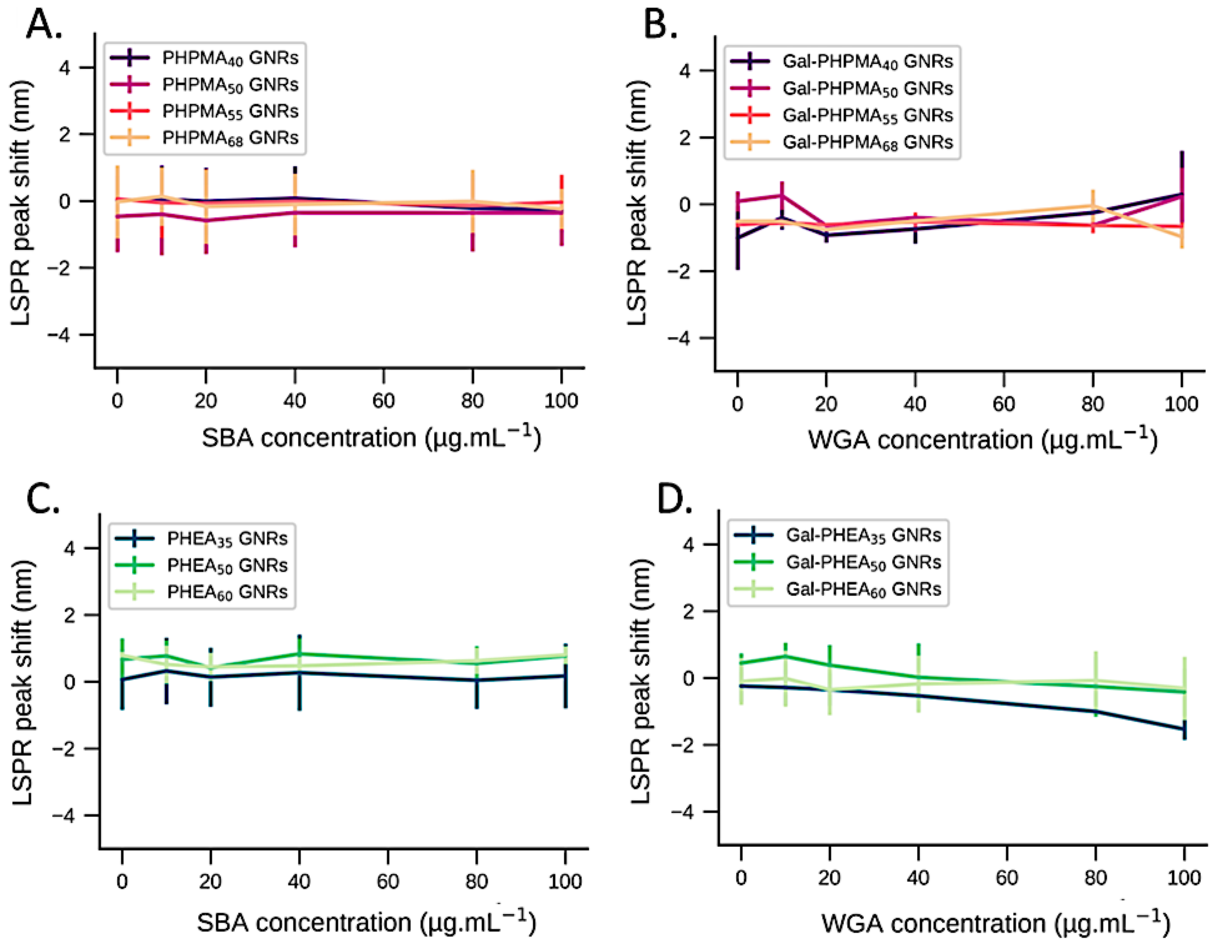

Figure S14. Control conditions of (Gal)-PHPMA and (Gal)-PHEA coated GNRs in human serum. LSPR peak shift of PHPMA (A) and PHEA (C) coated GNRs for different SBA concentrations as determined by UV-Vis spectroscopy in human serum. Same analysis was performed using Gal-PHPMA (B) and Gal-PHEA (D) coated GNRs for different WGA concentrations. All samples were analysed in duplicate every 30 mins for 2 hours.

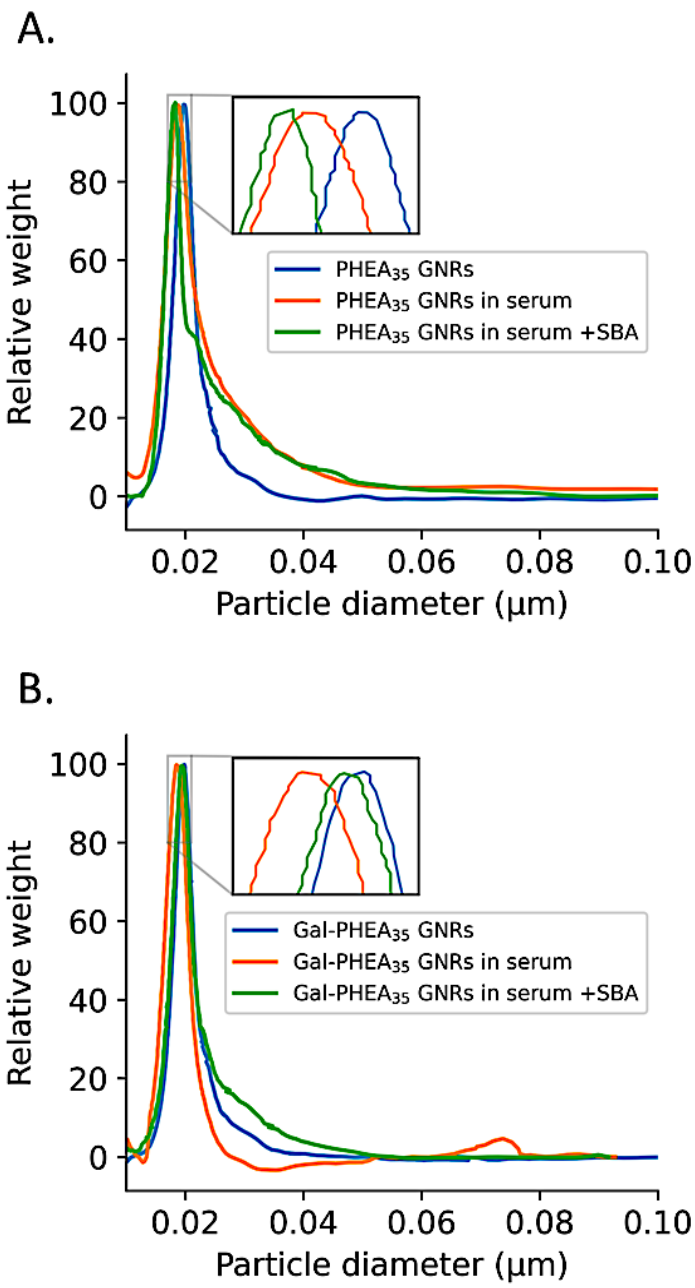

Figure S15. Differential centrifugal sedimentation analysis of (Gal)-$PHEA_{35}$ coated GNRs in buffer, human serum and serum spiked with 100 μg.mL^{-1} of SBA. Representative examples out of two replicates of relative weight in function of particle diameter (micrometers) of $PHEA_{35}$ GNRs (A) and Gal-$PHEA_{35}$ GNRs (B) in buffer (blue line), in serum (orange line) and after the addition of 100 μg.mL^{-1} of SBA (green line). Inset: zoomed view of the peaks.

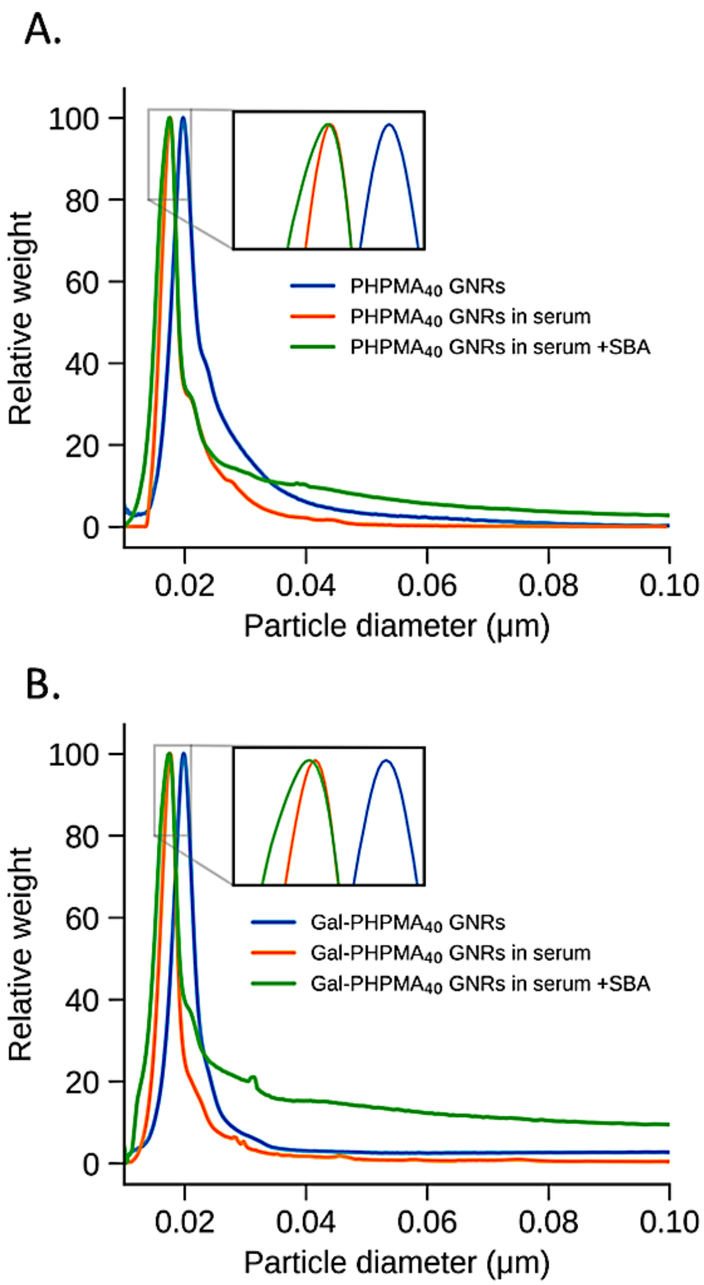

Figure S16. Differential centrifugal sedimentation analysis of (Gal)-PHPMA$_{40}$ coated GNRs in buffer, human serum and serum spiked with 100 µg.mL^{-1} of SBA. Representative examples out of two replicates of relative weight in function of particle diameter (micrometers) of PHPMA$_{40}$ GNRs (A) and Gal-PHPMA$_{40}$ GNRs (B) in buffer (blue line), in serum (orange line) and after the addition of 100 µg.mL^{-1} of SBA (green line). Inset: zoomed view of the peaks.

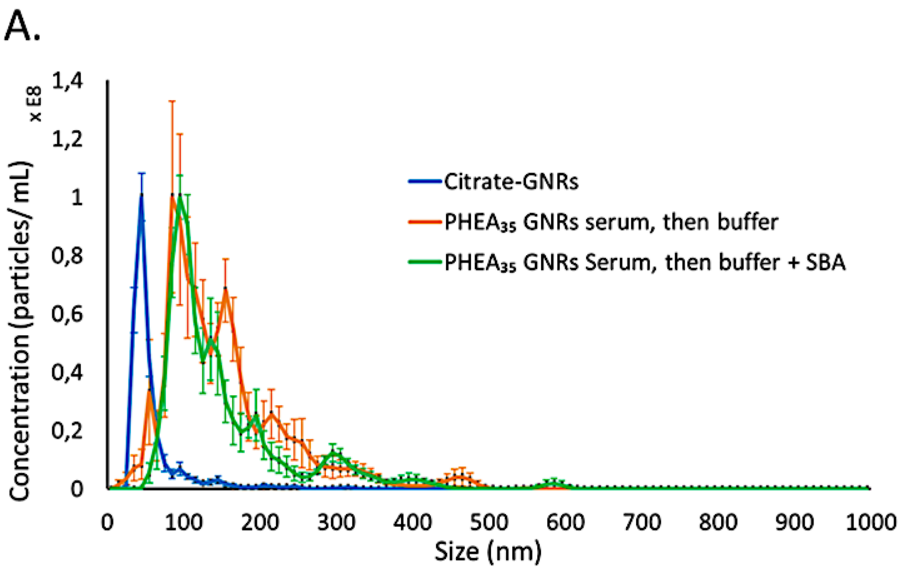

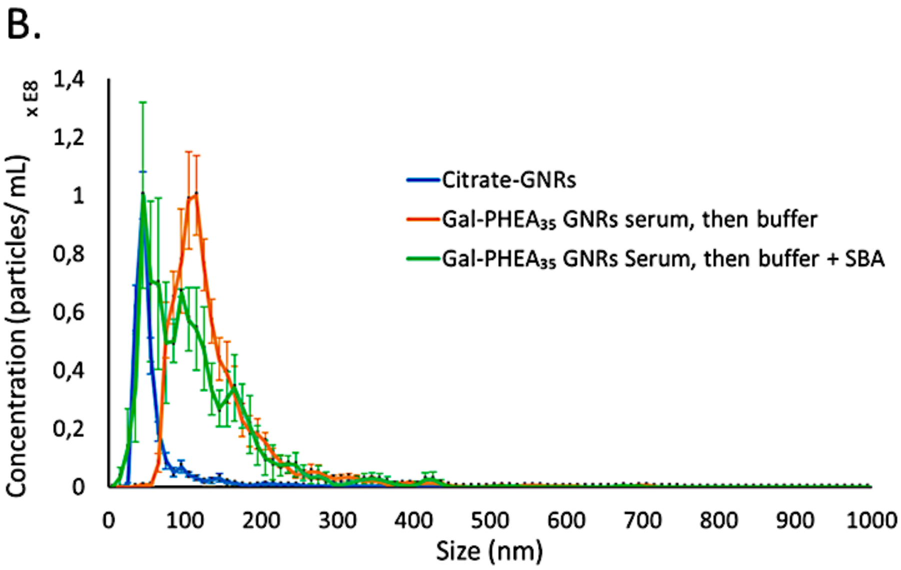

Figure S17. Particle number-based size distribution of (Gal)-$PHEA_{35}$ GNRs analysed by NTA. $PHEA_{35}$ GNRs (A) and Gal-$PHEA_{35}$ GNRs (B) were incubated in human serum to allow the formation of a biomolecular corona followed by washing the rods with buffer. Representative example of citrate-GNRs (blue line), samples in buffer after serum incubation (orange line) and after the addition of 100 µg.mL^{-1} of SBA (green line). For each step, samples were incubated for 2 hours at room temperature.

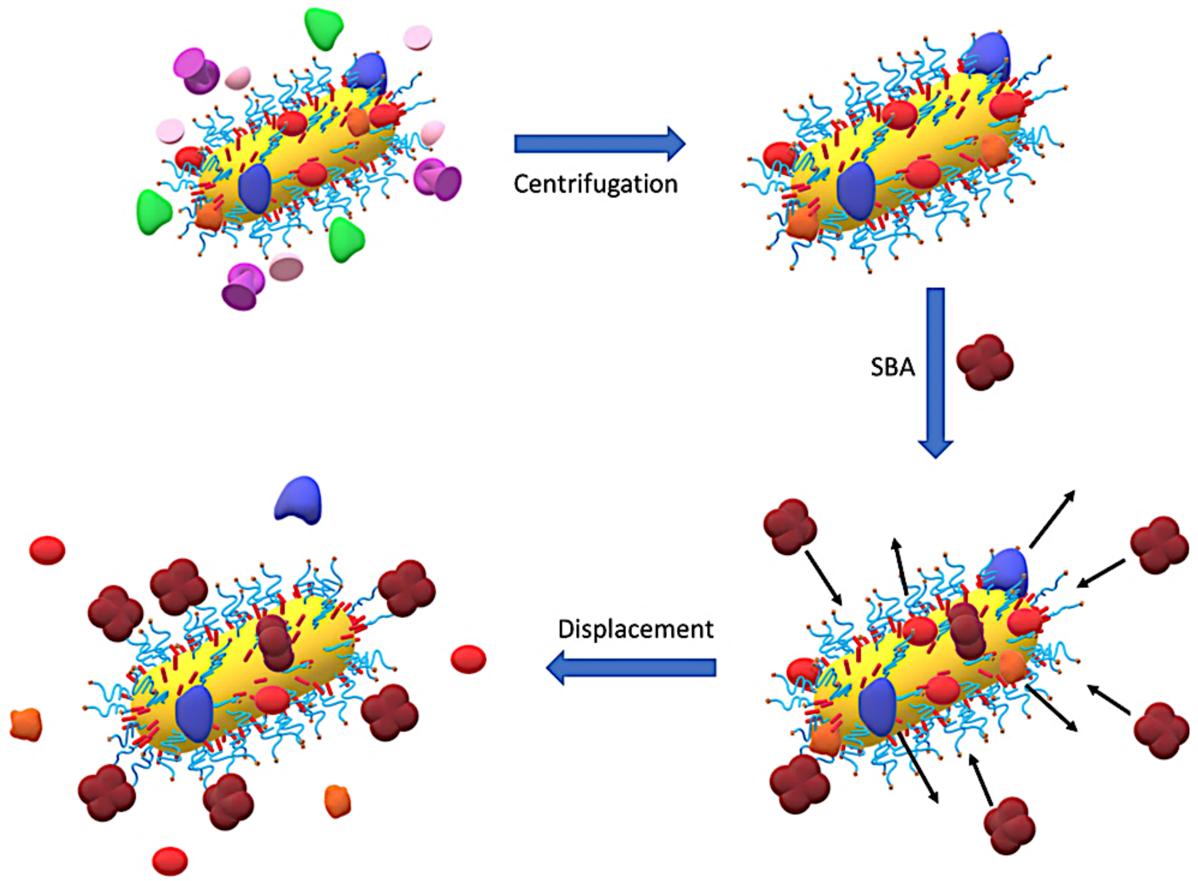

Figure S18. SBA binding to Gal-PHEA GNRs in human serum. Schematic illustration of the removal the “soft” corona following washing of the rods with buffer. Here, we hypothesised a replacement of the “hard” corona surrounding Gal-PHEA GNRs in serum spiked with SBA by the specific sugar-lectin interaction.
